# Distinct functional neutrophil phenotypes in sepsis patients correlate with disease severity

**DOI:** 10.3389/fimmu.2024.1341752

**Published:** 2024-03-08

**Authors:** Qingliang Yang, Jordan C. Langston, Roman Prosniak, Samantha Pettigrew, Huaqing Zhao, Edwin Perez, Hannah Edelmann, Nadia Mansoor, Carmen Merali, Salim Merali, Nathaniel Marchetti, Balabhaskar Prabhakarpandian, Mohammad F. Kiani, Laurie E. Kilpatrick

**Affiliations:** ^1^Department of Mechanical Engineering, College of Engineering, Temple University, Philadelphia, PA, United States; ^2^Department of Bioengineering, College of Engineering, Temple University, Philadelphia, PA, United States; ^3^Department of Thoracic Medicine and Surgery, Lewis Katz School of Medicine at Temple University, Philadelphia, PA, United States; ^4^Department of Biomedical Education and Data Science, Lewis Katz School of Medicine at Temple University, Philadelphia, PA, United States; ^5^Center for Inflammation and Lung Research, Lewis Katz School of Medicine at Temple University, Philadelphia, PA, United States; ^6^School of Pharmacy, Temple University, Philadelphia, PA, United States; ^7^Biomedical Technology, CFD Research Corporation, Huntsville, AL, United States

**Keywords:** sepsis, Organ-on-Chip, neutrophil heterogeneity, proteomics, neutrophil extracellular traps

## Abstract

**Purpose:**

Sepsis is a clinical syndrome defined as life-threatening organ dysfunction caused by a dysregulated host response to infection. Sepsis is a highly heterogeneous syndrome with distinct phenotypes that impact immune function and response to infection. To develop targeted therapeutics, immunophenotyping is needed to identify distinct functional phenotypes of immune cells. In this study, we utilized our Organ-on-Chip assay to categorize sepsis patients into distinct phenotypes using patient data, neutrophil functional analysis, and proteomics.

**Methods:**

Following informed consent, neutrophils and plasma were isolated from sepsis patients in the Temple University Hospital ICU (n=45) and healthy control donors (n=7). Human lung microvascular endothelial cells (HLMVEC) were cultured in the Organ-on-Chip and treated with buffer or cytomix ((TNF/IL-1β/IFNγ). Neutrophil adhesion and migration across HLMVEC in the Organ-on-Chip were used to categorize functional neutrophil phenotypes. Quantitative label-free global proteomics was performed on neutrophils to identify differentially expressed proteins. Plasma levels of sepsis biomarkers and neutrophil extracellular traps (NETs) were determined by ELISA.

**Results:**

We identified three functional phenotypes in critically ill ICU sepsis patients based on *ex vivo* neutrophil adhesion and migration patterns. The phenotypes were classified as: Hyperimmune characterized by enhanced neutrophil adhesion and migration, Hypoimmune that was unresponsive to stimulation, and Hybrid with increased adhesion but blunted migration. These functional phenotypes were associated with distinct proteomic signatures and differentiated sepsis patients by important clinical parameters related to disease severity. The Hyperimmune group demonstrated higher oxygen requirements, increased mechanical ventilation, and longer ICU length of stay compared to the Hypoimmune and Hybrid groups. Patients with the Hyperimmune neutrophil phenotype had significantly increased circulating neutrophils and elevated plasma levels NETs.

**Conclusion:**

Neutrophils and NETs play a critical role in vascular barrier dysfunction in sepsis and elevated NETs may be a key biomarker identifying the Hyperimmune group. Our results establish significant associations between specific neutrophil functional phenotypes and disease severity and identify important functional parameters in sepsis pathophysiology that may provide a new approach to classify sepsis patients for specific therapeutic interventions.

## Introduction

Sepsis is a major healthcare problem that accounts for approximately 20% of all global deaths. In the US, there are over 1.7 million sepsis cases/year and >250,000 deaths/year (1–3). Sepsis is a clinical syndrome, now defined as life-threatening organ dysfunction caused by a dysregulated host response to infection (4). Neutrophils are important contributors to the dysregulated immune response and play a critical role in sepsis-induced organ failure through interactions with the vascular endothelium resulting in barrier disruption and increased neutrophil trafficking into vital organs (5–8).

To date, treatment of sepsis is largely based on supportive care and there are no drugs available that target immune cell dysregulation. Drug development has been hindered for multiple reasons including limited translation from rodent models to patient pathology, the complexity of the underlying pathophysiology, and importantly the heterogenous nature of sepsis (9–12). There is now a consensus that the host response to sepsis is highly diverse among patients, and this heterogeneity impacts immune function and response to infection (10–21). While neutrophils are critical to host defense, neutrophil dysregulation in sepsis may play a critical role in the course of organ damage through the release of inflammatory mediators, proteases, neutrophil extracellular traps (NETs) and reactive oxygen species (ROS), which can damage endothelial cells leading to multiple organ failure and increased mortality (6–8, 22). Conversely, immunosuppression and neutrophils with compromised neutrophil function have also been reported in sepsis patients (6–8, 20, 23, 24). Some sepsis patients develop a mixed status with characteristics of persistent inflammation and immunosuppression (25, 26). These diverse responses to infection may explain the inconsistency in response to immunomodulating treatments and failures of sepsis clinical trials. Thus, a single, standard treatment for the heterogeneous cohort of sepsis patients has proven to be problematic and underscores the importance of categorizing sepsis patients into distinct immune phenotypes for personalized medicine (10).

Omics, cell surface marker expression, immune cell profiles, and biomarker analysis have been utilized to classify the immune status of sepsis patients (10, 12, 14–18, 27–29). However, no studies have examined how these omic changes correlate with fundamental mechanisms of neutrophil-mediated damage in sepsis, specifically how heterogeneous neutrophil-endothelial cell interactions differentially impact vascular barrier disruption and neutrophil migration across the endothelium into vital organs. Investigating neutrophil function during sepsis and correlating it to phenotypic proteomic analysis, is critical not only for a comprehensive understanding of the underlying molecular expression within the cells but also for determining how these changes can significantly affect immune function. To develop more effective and targeted therapeutics, careful identification of distinct functional phenotypes of these immune cells is required for classification of patients.

To examine human neutrophil-endothelial interactions, we developed and validated a novel Organ-on-Chip assay (30–32). This microfluidic assay reproduces vascular networks on a chip in a 3D physiologically relevant *in vitro* system which can evaluate the entire neutrophil adhesion cascade including circulation, rolling, adhesion, and migration of neutrophils under physiologically realistic (e.g. topology and shear conditions) microvascular environments. Our Organ-on-Chip design permits analysis of differences in spatial and flow dependent adhesion over different shear rates and at vessel bifurcations (33). The adhesion pattern of neutrophils in our Organ-on-Chip device is similar to patterns observed *in vivo* and in previous studies, we demonstrated that in response to proinflammatory cytokine activation, human neutrophil adhesion to human endothelial cells was significantly increased and greatest in vessels under low shear stress and at vessel bifurcations with minimal adhesion in high shear regions demonstrating how flow conditions strongly influence neutrophil adhesion to endothelial cells in the microvasculature (32, 34–36). This enhanced adhesion was associated with cytokine-induced upregulation of adhesion molecule expression and a significant increase in neutrophil migration across human endothelial cells, mimicking processes observed *in vivo* during inflammatory events.

In this study, we employed our Organ-on-Chip technology to categorize critically ill septic patients into distinct functional neutrophil phenotypes based on *ex vivo* neutrophil adhesion and migration patterns across primary human lung microvascular endothelial cells (HLMVEC). The goal of this study was to test the hypothesis that discrete neutrophil phenotypes in sepsis patients impact their ability to interact with the vascular endothelium and traffic into critical organs and that these distinct phenotypes are associated with disease severity. Further, we employed proteomics to characterize these neutrophil functional phenotypes and to identify distinct *proteomic signatures* related to functional neutrophil phenotypes. Thus, we employed a synergistic combination of Organ-on-Chip and proteomics to identify functional neutrophil phenotypes that differentiated sepsis patients by important clinical parameters related to disease severity.

## Materials and methods

### Study approval

The study was approved by the Temple University Institutional Review Board (Temple University IRB protocol #24515) and conducted according to the ethical guidelines of the Declaration of Helsinki. Written informed consent was obtained from the patient or a legally authorized representative. Healthy adult donors were recruited through the Thrombosis Research Center Blood Program (Temple University IRB protocol #0377) and written informed consent was obtained from all study participants.

### Study design and enrollment

Patients admitted to the Temple University Hospital Medical ICU with the diagnosis of sepsis or septic shock were eligible to participate in this study. The inclusion criteria for sepsis patients was defined according to the Third International Consensus Definition for Sepsis (Sepsis-3) (4) and included patients between the ages of 18 and 88 years old. Based on this definition, patients with suspected infection and an acute increase of ≥ 2 SOFA (Sequential [Sepsis-related] Organ Failure Assessment) were eligible for enrollment. The qSOFA (Quick SOFA) criteria includes a respiratory rate of > 22/min, altered mental status, and systolic blood pressure of ≤ 100mm Hg. Septic shock was defined clinically as patients fulfilling the criteria for sepsis with persisting hypotension requiring vasopressors to maintain MAP ≥ 65mm Hg and having a serum lactate level >2 mmol/L (18 mg/dL) despite adequate volume resuscitation. Sepsis patients were excluded from the study if they were <18 years old or were diagnosed with retroviral and chronic inflammatory diseases or conditions that require the chronic use of non-steroidal anti-inflammatories or NSAIDs whose use of would interfere with our study of neutrophil function. Following informed consent, a single 10-15cc of blood sample was obtained. Patient demographic, source of infection, and laboratory and clinical data were collected.

### Neutrophil isolation

Neutrophils were isolated from heparinized blood samples from sepsis patients and deidentified healthy adult donors. Human neutrophils were isolated by standard techniques using ficoll-hypaque separation, dextran sedimentation, and hypotonic lysis to remove erythrocytes (37).

### Culture of human lung microvascular endothelial cells

Primary HLMVEC were purchased from Lonza (Basel, Switzerland). HLMVEC were cultured in the microvascular endothelial growth media (MV-EGM) and used between passages 1-3 following manufacturer’s instructions as we reported previously (36, 38).

### Neutrophil adhesion and migration studies using Organ-on-Chip

**Figure 1:**
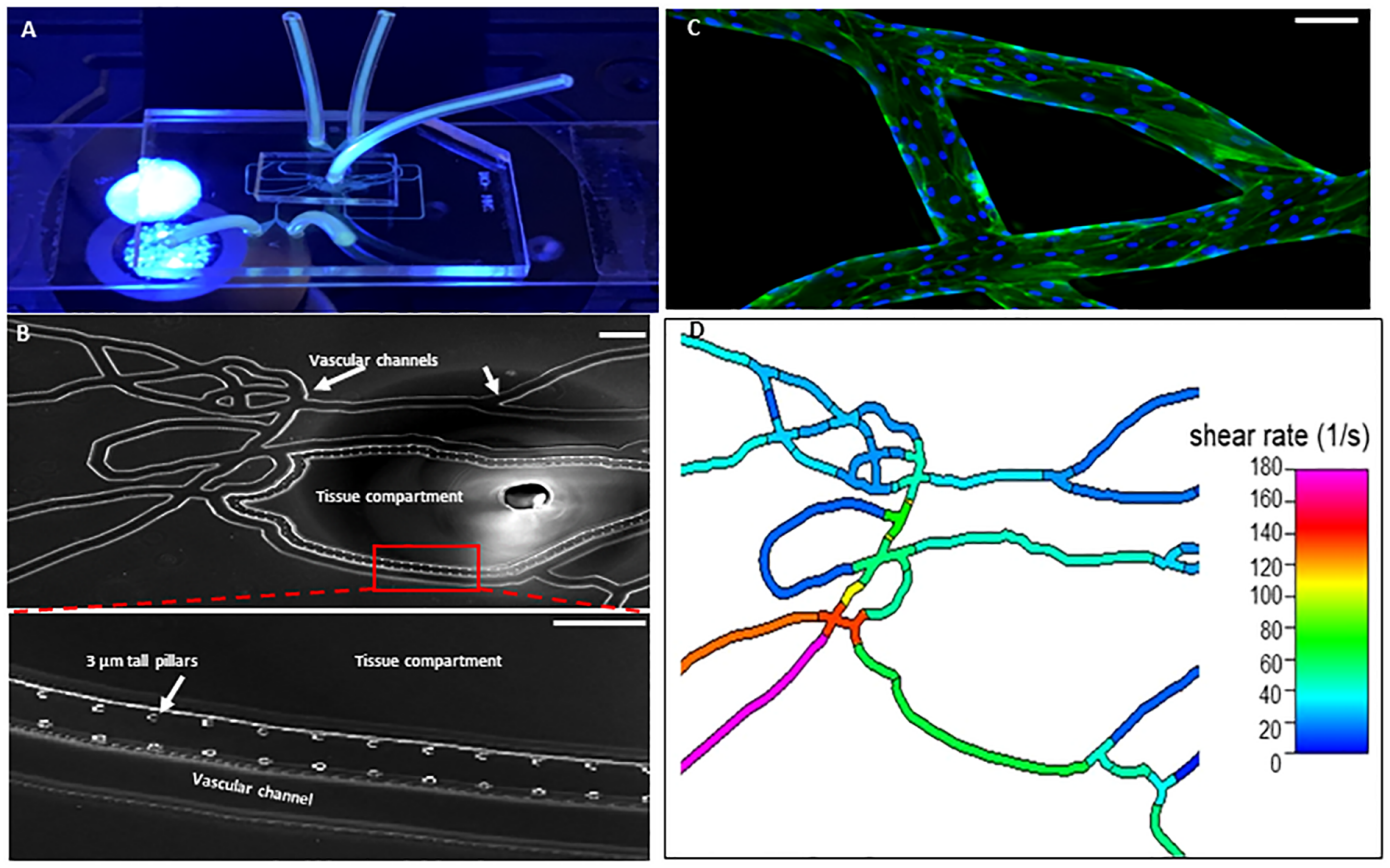
Organ-on-Chip Design **(A)** The image shows the vascular channel network and inlets and outlets with tubing inserted. **(B)** Bright field image shows the vascular channels and tissue compartment of the organ-on-chip. The vascular channels and tissue compartment are connected by 3 µm pores. **(C)** HLMVEC grow to confluent to cover the vascular channels. F-actin is labeled green with phalloidin and nuclei labeled blue with Hoechst 33342. (Scale bar = 100 µm). **(D)** Spatial variations in flow conditions in the vascular networks and at bifurcations showing shear rates in different vessel in the network in the organ-on-chip. Blue indicates a low shear rate and red a high shear rate. The effects of shear flow and vessel geometry on neutrophil adhesion and migration can be determined in this system.

As shown in **Figure 1**, our Organ-on-Chip (manufactured by SynVivo Inc., Huntsville, AL) is comprised of a 3D vascular compartment, reproduced from *in vivo* images, which is seeded with endothelial cells and a tissue compartment which can be filled with chemoattractants (e.g. fMLP); these two compartments are connected by 3 µm porous architecture, an optimum size for neutrophil migration (30, 31). HLMVEC were cultured in our Organ-on-Chip according to our published protocol (38). Prior to the injection of neutrophils, the Organ-on-Chip was treated for 4 hours under flow with buffer or cytomix (TNF-α (10 ng/mL) + IL-1β (5 ng/mL)+ IFN-γ (50 ng/mL)) to mimic inflammatory conditions (39–41). For cytomix experiments, fMLP (1 μM) was added to the tissue compartment as the chemoattractant. Neutrophils were fluorescently labeled using CFDA SE probe (Invitrogen, Eugene, OR) (32) and treated with buffer or cytomix for 15 minutes prior to injection into the vascular channels at a flow rate of 1 μL/min. Neutrophils were considered adherent if they did not move for 30 seconds. Neutrophil adhesion was determined at different shear rates and at vessel bifurcations over 60 minutes and the neutrophil adhesion map was obtained by scanning the entire network. A previously developed and published Computational Fluid Dynamics based model was employed to determine the shear stress in the different vessels in the vascular compartment (42). The number of neutrophils adherent at different shear rates were determined and plotted as shear rate vs. number of adherent neutrophils. Neutrophil migration into the tissue compartment was determined by quantifying the number of migrated neutrophils using timelapse imaging every 5 minutes for 60 minutes by scanning the tissue compartment. Nikon Elements software was used to collect and analyze the data.

### Neutrophil proteomic analysis

Isolated neutrophils were suspended in HBSS (2 x 10^6^ cells/ml), centrifuged and the cell pellets stored at -70°C prior to label-free global proteomic analysis. The proteomics analysis was performed in four neutrophil groups: Control, Hypoimmune, Hyperimmune, and Hybrid, with n=4 per group. Samples were prepared as follows: proteins were extracted by adding 6M of guanidium hydrochloride buffer and dilution buffer (25 mM Tris, 10% acetonitrile (pH 8.5). The proteins were digested with rLys-C mass spec grade for 4 hours at 37°C. A second digestion was achieved by overnight incubation with sequencing-grade modified Trypsin. The incubated solution was acidified and centrifuged at 4,500 g for 5 minutes. The supernatant consisting of peptides was loaded onto activated in-house cation stage tips (43–45). The peptides were eluted into six fractions using elution buffers and desalted as previously described (43, 44). Mass spectrometry (MS) analysis was performed on these desalted tryptic peptide fractions using the Q Exactive mass spectrometer (ThermoFisher Scientific, Waltam, MA, USA) (46–49). The fractions were loaded onto an Acclaim PepMap 100 pre-column (75 µm × 2 cm, ThermoFisher Scientific) and separated by Easy-Spray PepMap RSLC C18 column with an emitter (2 µm particle size, 15 cm × 50 µm ID, ThermoFisher Scientific) by an Easy nLC system with Easy Spray Source (ThermoFisher Scientific). To elute the peptides, a mobile-phase gradient was run using an increasing concentration of acetonitrile. The peptides were loaded in buffer A (0.1% (v/v) formic acid) and eluted with a nonlinear 145-min gradient as follows: 0–25% buffer B (15% (v/v) of 0.1% formic acid and 85% (v/v) of acetonitrile) for 80 min, 25–40% B for 20 min, 40–60% B for 20 min and 60–100% B for 10 min. The column was then washed with 100% buffer B for 5 min and re-equilibrated, 50% buffer B for 5 min and re-equilibrated with buffer A for 5 min. The flow rate was maintained at 300 nl/min. Electron spray ionization was delivered at a spray voltage of −1500 V. The MS/MS fragmentation was performed on the five most abundant ions in each spectrum using collision-induced dissociation with dynamic exclusion (excluded for 10.0 s after one spectrum), with automatic switching between the MS and MS/MS modes. The complete system was entirely controlled by Xcalibur software.

### Bioinformatic analysis

Mass spectra processing was performed with Proteome Discoverer (PD) version 2.5. The generated de-isotoped peak list was submitted to an in-house Mascot server 2.2.07 for searching against the Swiss-Prot database (Release 2013_01, version 56.6, 538,849 sequences), MS Amanda 2.0 database and Sequest HT database. Mascot, MS Amanda 2.0 and Sequest HT search parameters were set as follows: species, homo sapiens; enzyme, trypsin with maximal one missed cleavage; static modification, cysteine carbamidomethyl; 10 ppm mass tolerance for precursor peptide ions; 0.02 Da tolerance for MS/MS fragment ions. For dynamic modifications, we used oxidation/+15.995 Da (M) and N-terminal modification Met-loss/-131.040 Da (M). The grouped heat maps were generated using Proteome Discoverer.

Further analysis of the data exported to Microsoft Excel was performed using RStudio (v.4.1.2) bioinformatic analysis. Specifically, Pearson correlation coefficients (r) were calculated and transformed to Fisher z scale for a t-test with FDR correction to identify differentially expressed proteins (DEPs) or hits within neutrophil proteomes between groups. Bioconductor (v.3.14), within R, was used to analyze the protein lists via the BiocManager package. Proteins with a fold change>2 and a FDR-adjusted p<0.01 were characterized as upregulated, while proteins with a fold change<0.5 and a FDR-adjusted p<0.01 were downregulated. FDR was controlled using Benjamini-Hochberg procedure (50). Volcano plots highlighting neutrophil differential protein expression were generated using ggplot2 in R. The identified proteins from the analysis were compared for fold changes in order to identify the top 10 proteins with the maximal changes. This was performed by including proteins that had a fold change > 2 or < 0.5, a p < 0.05, for Hyperimmune/Hypoimmune expression in at least 2 of the 4 samples analyzed across the phenotypes. Heatmaps were plotted in RStudio using the heatmap.2 function as part of the gplots package. Venn diagrams highlighting the number of unique and common proteins shared amongst the groups were produced using the venn.diagram function as part of the venn diagram package. Hierarchical sample clustering was done by using the complete linkage method and Euclidean distance metric. Data was scaled before plotting.

### Plasma biomarkers

Plasma biomarkers were measured by ELISA (R & D Biosystems, Minneapolis, MN) for IL-6 (cat# D6050), IL-8/CXCL8 (cat# D8000C), ICAM-1 (cat# DY720-05), vWF-A2 (cat# DY2764-05), and Angiopoietin-2 (cat# DY623) according to the manufacturer’s instructions.

### NETs

Plasma levels of MPO : DNA complexes were measured by ELISA as a marker of plasma NETs (51). MPO : DNA complexes were determined by sandwich ELISA using anti-MPO (Biorad, cat# 0400-0002) as the capture antibody and HRP- conjugated anti-DNA (Cell Death Detection Kit, Cat# 11544675001 Roche) as the specific detection antibody (52).

### Statistical analysis

Adhesion and migration data are presented as mean ± SEM. Statistical significance was determined by one-way or two-way analysis of variance (ANOVA) with Tukey Kramer *post hoc* using SigmaPlot software. Patient characters continuous variables are reported as mean ± SEM (or SD) and median with IQR. Categorical variables were summarized using counts and percentages. Chi-square or ANOVA tests were conducted to determine statistical significance of the variables from the different phenotypes. For data that is not normally distributed, transformation such as logarithm was used. Canonical discriminate analysis was conducted to classify sepsis patient phenotypes by using continuous variables a) neutrophil adhesion ± cytomix at different shear rates (<15, 15-30, 30-60, 60-150 s-1) and at bifurcations, and b) neutrophil migration ± cytomix over 60 min (10,15, 30, 60 min). All tests resulting in *p*<0.05 were considered statistically significant.

## Results

### Functional studies reveal three neutrophil phenotypes

To investigate neutrophil functional responses in critically ill sepsis patients, following informed consent, neutrophils were obtained from 45 patients in the Temple University Hospital Medical ICU, who were diagnosed with sepsis. Only sepsis patients who required ICU care were enrolled in the study. The general characteristics of the sepsis ICU patients are shown in **Table 1**. *E. coli* bacteria was the most common bacterial strain isolated from these patients and the source of sepsis was predominantly pulmonary in nature. This patient population had an average ICU stay of 13 days, 56% required mechanical ventilation, 42% were in septic shock, and had an ICU mortality rate of 43%.

**Table 1:** Patient demographics.

Patient Characteristics	ICU Sepsis Patients (N=45)
Age (Mean (range))	56 (24-88)
Gender N (% male)	24 (53%)
Ethnic Group N (%)	
Caucasian	18 (40%)
Black	14 (31%)
Hispanic	10 (22%)
Asian	3 (7%)
Type of Infection N (%)	
Gram negative bacteria	15 (33%)
Gram Positive bacteria	10 (22%)
Mixed organisms	10 (22%)
Fungal	2 (4%)
Organism negative	8 (18%)
Source of Infection N (%)	
Pulmonary	24 (53%)
UTI	8 (18%)
Abdomen	6 (13%)
Blood	5 (11%)
Soft Tissue	2 (4%)
Laboratory Values (Mean ± SD)	
WBC ×10^9^/L	15 ± 5
Neutrophils ×10^9^/L	6 ± 4
Illness Severity	
qSOFA Score (Mean ± SD)	2 ± 1
Glasgow Coma Score (Mean ± SD)	9 ± 5
Mechanical Ventilation N (%)	25 (56%)
Septic Shock N (%)	19 (42%)
Outcomes	
ICU-length of stay (days) (Mean ± SD)	13 ± 12
ICU Mortality N (%)	19 (43%)

UTI, Urinary Tract Infection; WBC, white blood cells; qSOFA, quick sequential organ failure assessment.

Neutrophils isolated from sepsis patients (n=45) and healthy control donors (n=7) were examined for their response to a mix of proinflammatory cytokine (cytomix: TNF-α/IL-1β/IFN-γ) stimulation as compared to buffer-treated cells. Using the Organ-on-Chip assay, the effect of cell activation *ex vivo* was measured by neutrophil adhesion to HLMVEC in the vascular compartment and migration into the tissue compartment of the Organ-on-Chip (**Figure 1**, **Supplementary Video 1**).

**Figure 2:**
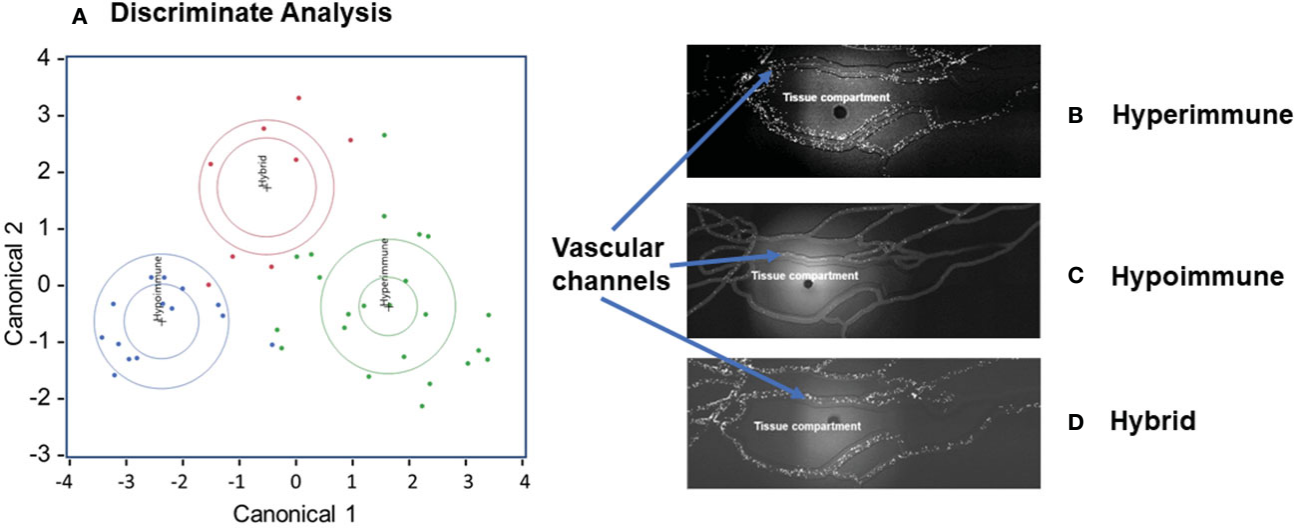
Neutrophil adhesion and migration patterns identify distinct neutrophil functional phenotypes. **(A)** Discriminant analysis of neutrophil adhesion and migration patterns to identify different neutrophil functional phenotypes. Based on distinct adhesion and migration responses to cytomix activation, three different phenotypes were identified, and labeled as Hyperimmune, Hypoimmune and Hybrid. Continuous variables used for Discriminant Analysis include neutrophil adhesion ± cytomix at different shear rates (<15, 15-30, 30-60, 60-150 s^-1^) and at bifurcations, and neutrophil migration ± cytomix over 60 min (10,15, 30, 60 min). **(B)** Hyperimmune: Representative functional response of Hyperimmune neutrophils to cytomix in the organ-on-chip demonstrating increased neutrophil adhesion in the vascular channels and increased migration across human endothelial cells into the Tissue Compartment, **(C)** Hypoimmune: Representative functional response of Hypoimmune neutrophils to cytomix in the organ-on-chip demonstrating decreased neutrophil adhesion in the vascular channels and decreased migration across human endothelial cells into the Tissue Compartment and **(D)** Hybrid: Representative functional response of Hybrid neutrophils to cytomix in the organ-on-chip demonstrating increased neutrophil adhesion in the vascular channels and decreased migration across human endothelial cells into the Tissue Compartment.

There were significant differences in the characteristics of neutrophil adhesion and migration among sepsis patients when treated with cytomix ranging from enhanced adhesion and migration as compared to buffer-treated cells to neutrophils that were unresponsive to cytomix activation with limited adhesion and migration. Using Discriminant Analysis, we classified neutrophil phenotypes based on continuous variables which included a) neutrophil adhesion ± cytomix at different shear rates (<15, 15-30, 30-60, 60-150 s^-1^) and at bifurcations, and b) neutrophil migration ± cytomix over 60 min (10,15, 30, 60 min intervals). This analysis of the distribution of neutrophil functional adhesion at different shear rates and migration patterns identified three different patient groups (**Figure 2A**) corresponding to three distinct clusters of neutrophil responses to cytomix activation. **Figures 2B-D** shows representative images of the three different neutrophil adhesion and migration patterns in the Organ-on-Chip. These distinct phenotypes were characterized as a Hyperimmune phenotype (N=23, **Figure 2B**) with increased adhesion and migration in response to cytomix activation as compared to buffer-treated, a Hypoimmune phenotype (N=14, **Figure 2C**) with little or no response to cytokine activation in adhesion or migration patterns, and a third Hybrid phenotype (N=8, **Figure 2D**) with increased adhesion in response to cytomix activation but little or no migration as compared to buffer-treated neutrophils. The results indicated that 41 patients (91.1%) were correctly classified in this analysis.

**Figure 3:**
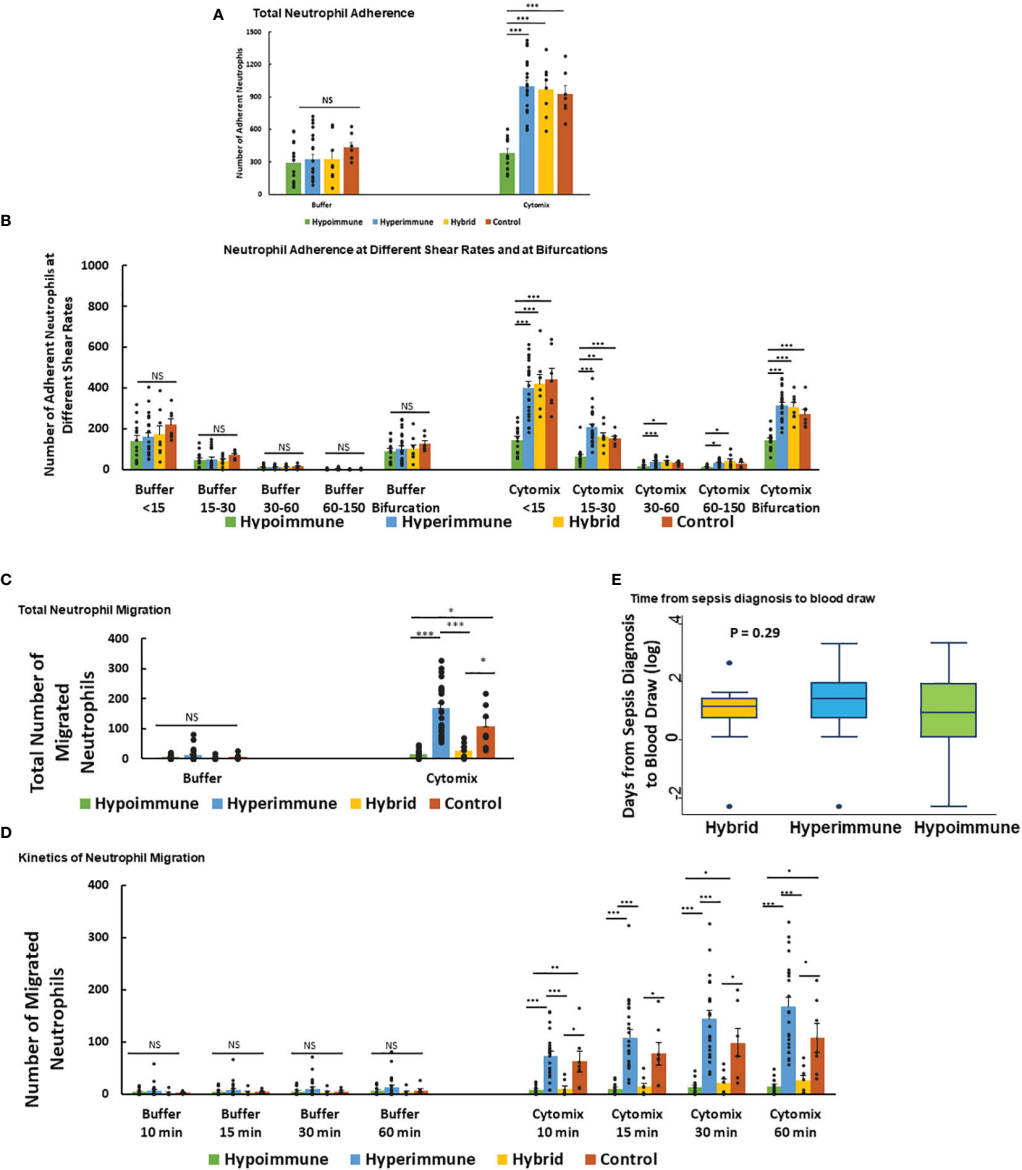
Neutrophil adhesion and migration in response to cytomix **(A)** Total neutrophil adhesion in organ-on-chip in the different sepsis neutrophil phenotypes and healthy controls in response to buffer or cytomix activation. **(B)** Adhesion of different neutrophil phenotypes at various shear rates and at bifurcations in the organ-on-chip. **(C)** Total neutrophil migration across HLMVEC into the tissue compartment following cytomix activation in response to fMLP. **(D)** The kinetics of sepsis patient neutrophil migration at different time points over a 60 min observation period. **(A-D)** Values are Mean ± SEM, *P<0.05, **P<0.01, ***P<0.001 by ANOVA. **(E)** Boxplot of time from sepsis diagnosis to blood draw in the Hyperimmune, Hypoimmune and Hybrid patient groups. P=0.29 by one way ANOVA.

Neutrophil adhesion to vascular endothelium under shear flow precedes neutrophil migration and is a key regulator of the inflammatory response. Analysis of total neutrophil adhesion in the Organ-on-Chip (**Figure 3A**) demonstrated phenotype-specific responses to cytomix activation. The Hyperimmune group demonstrated a 3-fold increase in neutrophil adhesion in response to cytomix as compared to buffer-treated neutrophils (P<0.001). The Hybrid group also demonstrated a similar increase in adhesion in response to cytomix (P<0.001). In contrast, the Hypoimmune group showed no significant differences between buffer and cytomix-treated groups in total neutrophil adhesion, and the Hypoimmune cytomix-treated neutrophil adhesion was significantly reduced as compared to Hyperimmune and Hybrid groups, as well as healthy controls (P<0.001). There were no significant differences in neutrophil adhesion between the three patient groups and controls in the absence of activation (i.e. buffer-treated) at the different shear rates (**Figure 3B**). Following cytomix activation, however, there was increased adhesion of neutrophils in the Hyperimmune and Hybrid groups at the shear rates <15 s^-1^, 15-30 s^-1^, 30-60 s^-1^ and at bifurcations as compared to the Hypoimmune group (P<0.05).

Neutrophil migration across HLMVEC exhibited distinct patterns in response to cytomix activation in the different phenotypes (**Figure 3C**). The Hyperimmune group demonstrated increased cytomix-induced neutrophil migration into the tissue compartment as compared to buffer-treated cells (P<0.001). In contrast, the Hypoimmune group showed no significant differences in migration between buffer and cytomix-treated migration (P=NS). The Hybrid group showed a small but statistically significant increase in neutrophil migration after cytomix treatment compared to buffer-treated (P<0.05), but this cytomix-induced increase in migration was significantly less than either the control or Hyperimmune response (P <0.05, **Figure 3C**). Following cytomix treatment, the Hyperimmune group migration was significantly increased compared to Hypoimmune and Hybrid groups (P<0.001) at all time points (10, 15, 30 and 60 minutes, **Figure 3D**). The Hybrid and Hypoimmune groups were also significantly decreased as compared to controls at all time points. Conversely, the Hybrid and the Hypoimmune groups were not significantly different from each other at any of the time points. There were no significant differences between any of the groups in the absence of stimuli (**Figure 3D**). Thus, we found significant differences in the characteristics of *ex vivo* neutrophil adhesion and migration patterns among critically ill ICU sepsis patients.

We next determined whether the timing of the blood draw from the diagnosis of sepsis affected the neutrophil phenotypes. We found that there were no significant differences among the patient groups in the timing of blood draws from the time of sepsis diagnosis indicating the phenotypes were not differentiated according to timing of sample acquisition (**Figure 3E**). We further determined whether the presence of bacteremia altered the neutrophil phenotypes in sepsis patients. Analysis of patient’s bacterial cultures found 13% of patients in the Hyperimmune (n= 23 patients), 50% in the Hybrid group (n=8 patients), and 21% in the Hypoimmune group (n= 14 patients) had positive blood cultures. There were no statistically significant differences in the presence of bacteremia among the three groups using Fisher’s Exact test (P=0.11). Thus, neither the timing of the blood draw or the presence of bacteremia impacted the functional phenotyping of neutrophils from ICU sepsis patients.

### Functional neutrophil phenotypes are associated with distinct proteomic signatures

**Figure 4:**
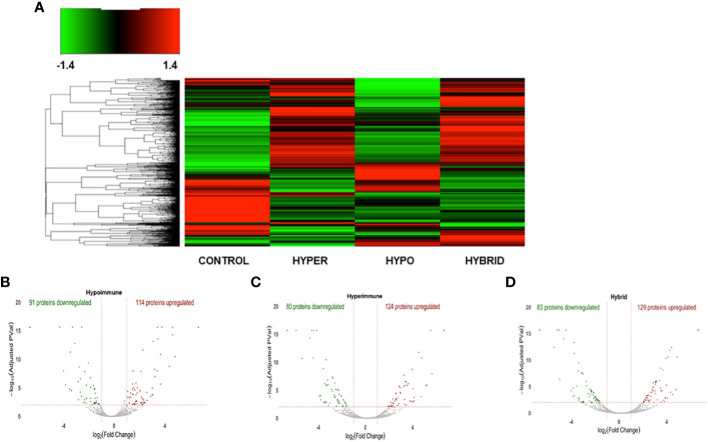
The three sepsis patient phenotypes have unique proteomic signatures. **(A)** Heatmap of neutrophil proteins **(B)** Volcano Plot of Hypoimmune phenotype as compared to healthy controls, **(C)** Volcano Plot of Hyperimmune phenotype as compared to healthy controls, **(D)** Volcano Plot of Hybrid phenotype as compared to healthy controls. For all three phenotypes (3B-3D), the red dots represent upregulated proteins, the green dots represent downregulated proteins, and the gray dots are proteins that are not significantly different from healthy control samples.

We next determined whether the significant differences in neutrophil function in the phenotypes were associated with altered protein expression. Proteomic analysis was done on freshly isolated neutrophils in the absence of exogenous stimuli to ascertain whether there were intrinsic alterations in these neutrophils. Both unique and common proteins were identified among the different patient groups and healthy adult controls. When patient neutrophils were grouped according to the functional phenotypes, there were significant differences in protein expression in the three sepsis groups as compared to healthy control neutrophils (**Figure 4A**). Further, there were distinct difference among the sepsis phenotypes particularly in the protein expression patterns between the Hyperimmune and Hypoimmune neutrophils. Volcano plots (**Figures 4B-D**) highlight the upregulated, downregulated, and unchanged expression in a large number of proteins in the different patient groups as compared to healthy controls.

**Figure 5:**
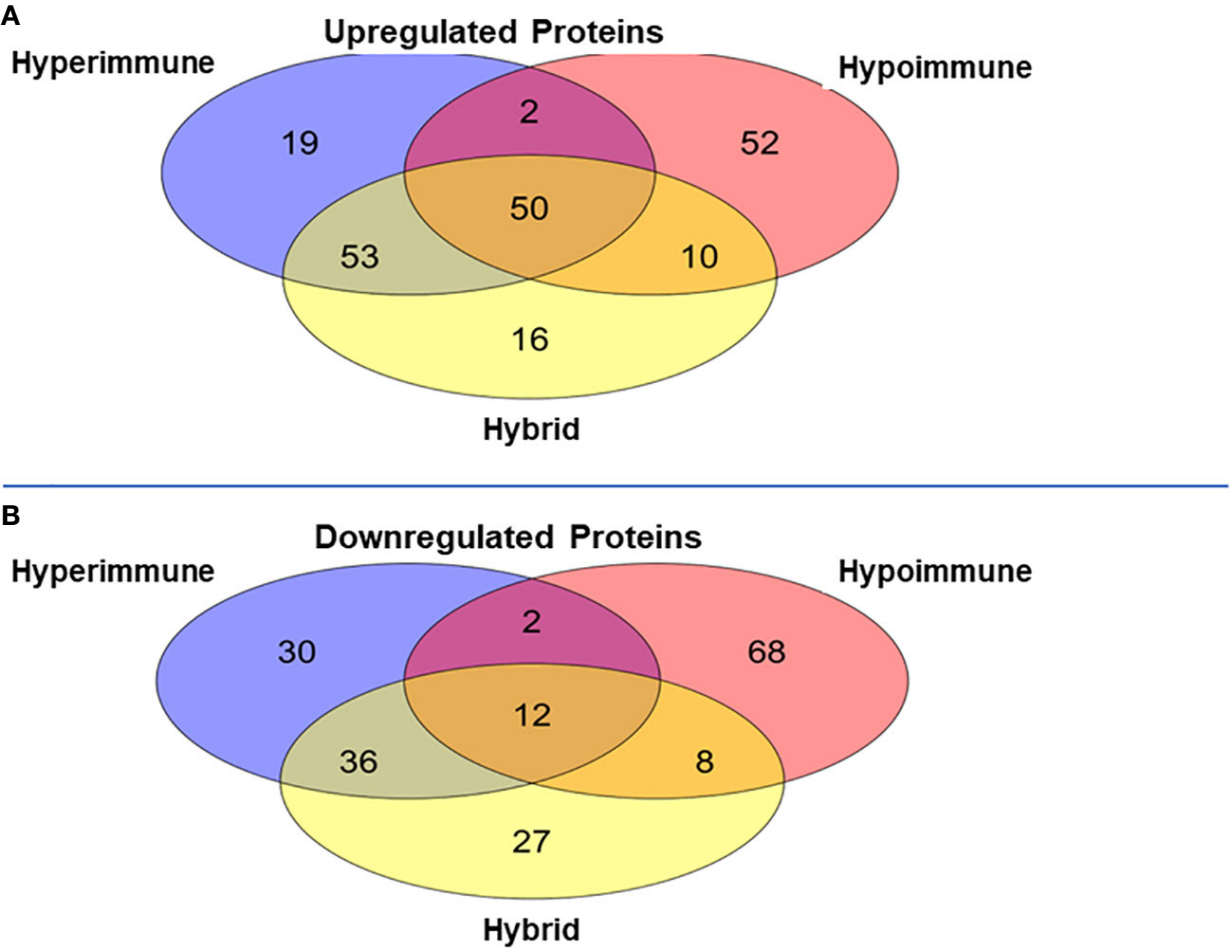
Venn Diagrams of protein expression changes in the neutrophils from the three sepsis patient phenotypes as compared to controls. **(A)** Common and unique upregulated proteins in the neutrophils from the three sepsis patient phenotypes. **(B)** Common and unique downregulated proteins in the neutrophils from the three sepsis patient phenotypes.

Venn diagrams revealed significant differences among the proteomics of different phenotypes (**Figure 5**). There were 50 proteins commonly upregulated in the three sepsis phenotypes as compared to controls (**Figure 5A**, **Supplementary Table 1**). The Hypoimmune group had the highest number of uniquely expressed proteins (52) as compared to controls, while the Hyperimmune and Hybrid groups had 19 and 16 proteins, respectively (**Supplementary Table 2**). The Hypoimmune group only had two upregulated proteins shared with the Hyperimmune group, and only ten upregulated proteins shared with the Hybrid group. In contrast, there were 53 upregulated proteins shared between the Hyperimmune and Hybrid groups indicating more protein overlap between the Hyperimmune and Hybrid group than with the Hypoimmune group (**Supplementary Table 3**).

There was a significant number of proteins that were differentially downregulated in the three sepsis phenotype groups as compared to controls (**Figure 5B**). There were 12 proteins commonly downregulated in the three sepsis groups as compared to controls (**Supplementary Table 1**). Similar to the upregulated proteins, the Hypoimmune group had the highest number of unique downregulated proteins (68 proteins) as compared to the Hyperimmune (30 proteins) and Hybrid (27 proteins) groups (**Supplementary Table 2**). Similarly, the Hypoimmune group had only two downregulated proteins shared with the Hyperimmune group, and eight downregulated proteins shared with the Hybrid group. In contrast, the Hyperimmune and Hybrid group shared 36 downregulated proteins (**Supplementary Table 3**). These proteomic results are consistent with the significant functional differences observed between the Hypoimmune group compared to the Hyperimmune and Hybrid groups. These studies indicate that neutrophil functional differences in the different phenotypes are associated with altered protein expression in the different groups.

**Figure 6:**
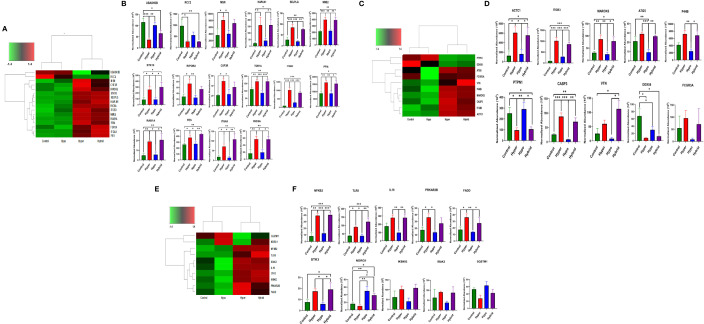
Differential expression of neutrophil adherence proteins in sepsis phenotypes **(A)** A clustered heatmap showing expression of neutrophil adherence associated proteins in control, Hypoimmune, Hyperimmune and Hybrid groups. The color key at the top left indicates whether protein expression was greater or less than the mean. **(B)** Quantitation of the individual adherence associated proteins. **(C)** A clustered heatmap showing expression of neutrophil cytoskeleton associated proteins. **(D)** Quantitation of the individual cytoskeleton associated proteins. **(E)** A clustered heatmap showing expression of neutrophil defense associated proteins. **(F)** Quantitation of the individual defense associated proteins. Values are Mean ± SEM, *P<0.05, **P<0.01, ***P<0.001, n=4/group.

To provide further mechanistic insight into the observed functional differences between the neutrophil phenotypes, we constructed heatmaps of differentially expressed proteins associated with important aspects of neutrophil function in sepsis, such as adhesion, cytoskeleton and host defense, to investigate commonality between the biological replicates across the phenotypes and determine whether there were unique proteomic signatures in the different groups. The heatmaps are clustered by phenotype which indicates that protein expression of the Hyperimmune and Hybrid phenotype neutrophils cluster together and are significantly different from the Hypoimmune phenotype which clusters with healthy controls (**Figure 6**). While the Hypoimmune group clustered with the controls, there were still significant differences in protein expression between the two groups. Further analysis (**Figures 6A, B**) underscores the significant upregulation of adherence associated proteins in the Hyperimmune and Hybrid phenotypes, which had significantly increased *ex vivo* neutrophil adhesion to endothelial cells (See **Figures 2**, **3**). A comparison of these proteins from the different phenotypes (**Figure 6B**) demonstrates that expression of proteins associated with neutrophil adhesion were significantly upregulated in the Hyperimmune phenotype as compared to controls and the Hypoimmune phenotype (P<0.05). Of interest, SELPLG, STK10, TOR1A, ITGB3, PPIA, RAB1A, FES, ITGAX, RIC8A, and NME2 expression was significantly increased in the Hyperimmune and Hybrid phenotypes as compared to the Hypoimmune phenotype (P<0.05) and may serve as potential biomarkers to differentiate the phenotypes. Conversely, UBASH3B (a protein tyrosine phosphatase and member of the TULA family) was significantly downregulated in the Hyperimmune phenotype as compared to the Hypoimmune phenotype (P<0.05). UBASH3B can have a suppressor role of host responses to pathogens (53).

Similar alterations in protein expression were observed in proteins associated with the cytoskeleton (**Figures 6C, D**). Expression of proteins important for neutrophil migration and adhesion including MARCKS (54), ITGB3 (55), and P4HB (PDI) (56) were significantly increased in the Hyperimmune and Hybrid phenotypes as compared to the Hypoimmune and control phenotypes (P<0.05). In contrast, the protein tyrosine phosphatase non-receptor type 1 (PTPN1/PTP1B) exhibited decreased expression in the Hyperimmune and Hybrid phenotypes as compared to the Hypoimmune and Control phenotypes (P<0.05). Deficiency of this protein has been shown to exacerbate inflammation and increase neutrophil trafficking *in vivo* (57). Expression of defense proteins were also significantly different with similar clustering of the controls with the Hypoimmune group and the Hyperimmune clustering with the Hybrid phenotype (**Figures 6E, F**). In the Hyperimmune and the Hybrid phenotype, as compared to controls and the Hypoimmune phenotype, there is significant increase in the expression of the NFKB2, a subunit of NFκB and an important regulator of the inflammatory response, as well as increased expression of proinflammatory mediators including IL-18, the toll-like receptor TLR8, and STK3. Thus, we demonstrate significant differences in expression among the patient groups in neutrophil proteins involved in critical aspects of the septic response. Proteomic profiling of neutrophils obtained from septic patients recapitulated a number of molecules regulating functional attributes of the different neutrophil phenotypes and indicated there were significant intrinsic differences in protein expression among these functional groups. These studies demonstrate that there are unique “proteomic signatures” for the Hyperimmune, Hypoimmune and Hybrid neutrophil phenotypes.

### Neutrophil phenotypes correlate with disease severity

**Figure 7:**
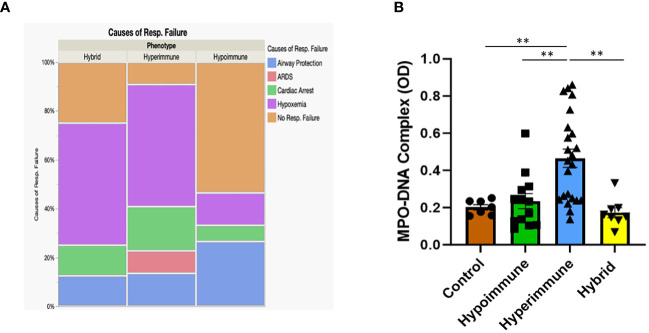
**(A)** Mosaic plot comparing the causes of respiratory failure in the Hypoimmune, Hyperimmune and Hybrid patient groups. **(B)** Plasma levels of MPO-DNA in healthy controls, Hypoimmune, Hyperimmune and Hybrid groups. Values are Mean ± SEM, (N= 8-23) **p<0.01.

When patients were grouped according to their functional neutrophil phenotype, there were no significant differences between the patient groups in their ages, gender, ethnicity, type of infection, source of infection and other clinical variables (**Supplementary Table 4**). Further, we found no significant differences in clinical assessments of disease severity (i.e., qSOFA and Glasgow Coma Scores) between the Hyperimmune, Hypoimmune and Hybrid groups (**Table 2**). However, those in the Hyperimmune and Hybrid groups had higher oxygen requirements and were more likely to require mechanical ventilation compared to the Hypoimmune group (**Table 2**). Consistent with these findings, hypoxemia and ARDS were the greatest cause of respiratory failure in the Hyperimmune group (59%) and Hybrid (50%) groups as compared to the Hypoimmune group (13%, P<0.05) (**Figure 7A**) . The Hyperimmune group had a significantly longer length of stay in the ICU (18.3 ± 3.1 days) as compared to the Hypoimmune and the Hybrid groups (6.9 ± 1.8 and 8.3 ± 2.4 days, respectively, P<0.01) (**Table 2**). Thus, in sepsis patients in the ICU, we identified associations between neutrophil phenotypes and important clinical parameters such as severity of hypoxemia, mechanical ventilation requirements, and ICU length of stay.

**Table 2:** Disease severity by phenotype.

Sepsis Phenotype	Hypoimmune	Hyperimmune	Hybrid	P value
Number of Subjects	N=14	N=23	N=8	
Illness Severity				
qSOFA Score (Mean ± SD)	1.4 ± 0.7	1.7 ± 0.6	1.5 ± 1.1	NS
Glasgow Coma Score (Mean ± SD)	10.5 ± 4.7	8.7 ± 4.8	9.3 ± 3.9	NS
FiO_2_ (%) ((Mean ± SD)	40.3 ± 24.8	57.7 ± 27.9	67.6 ± 33.7	P<0.05
Mechanical Ventilation N (%)	28.6%	69.6%	62.5%	P<0.05
Septic Shock N (%)	28.6%	47.8%	50.0%	NS
Outcomes				
ICU-Length of Stay (days) (Mean ± SD)	6.9 ± 6.6	18.3 ± 14.7	8.3 ± 6.8	P<0.01
ICU Mortality N (%)	5 (36%)	11 (50%)	3 (38%)	NS

qSOFA, quick sequential organ failure assessment; FiO_2_, fraction of inspired oxygen.

### Plasma biomarkers associated with specific neutrophil phenotypes

We next determined whether there were plasma biomarkers that differentiated the patient groups. In addition to functional differences in these different phenotypes, we found a significant difference in the number of circulating neutrophils. While there were no significant differences in the total WBC count between the patient groups (**Table 3**), there was a 2-fold increase in the number of neutrophils isolated from the Hyperimmune group as compared to the Hypoimmune or Hybrid groups (P<0.01). This higher number of circulating neutrophils was not related to increased release of immature neutrophils as there were no significant differences in blood levels of bands among the groups (**Table 3**).

**Table 3:** Plasma biomarkers in sepsis phenotypes.

Sepsis Phenotype	Hypoimmune	Hyperimmune	Hybrid	Control	P value
Number of Subjects	N=14	N=23	N=8	N=8	
Laboratory Values					
WBC ×10^9^/L	13 ± 6	16 ± 4	16 ± 8	NA	NS
Neutrophils ×10^9^/L	4 ± 2	8 ± 4	4 ± 2	NA	P<0.01 Hyperimmune vs. Hypoimmune and Hybrid
Bands (%)	1 ± 2	3 ± 12	1 ± 2	NA	NS
Plasma Markers of Endothelial Damage (Mean ± SEM)					
vWF (pg/ml)	10753 ± 1340	8276 ± 1363	6699 ± 752	3221 ± 539	P<0.01 vs. control
ICAM-1 (ng/ml)	145 ± 30	178 ± 36	192 ± 52	42 ± 3	P<0.001 vs. control
Angiopoietin (pg/ml)	5441 ± 1054	6526 ± 1631	8563 ± 4755	474 ± 37	P<0.001 vs. control
Plasma Cytokine Biomarkers (Mean ± SEM)					
IL-6 (pg/ml)	191 ± 96	184 ± 46	332 ± 107	2 ± 0.3	P<0.02 vs. control
IL-8 (pg/ml)	105 ± 49	207 ± 80	393 ± 311	2 ± 0.4	P<0.02 vs. control

We next assessed whether, similar to alterations in systemic neutrophil numbers, plasma biomarkers associated with sepsis could differentiate sepsis patients with distinct functional neutrophil phenotypes. We focused on sepsis biomarkers that are associated with neutrophil-endothelial interactions and vascular barrier disruption (58–60). Plasma markers of endothelial cell damage (sICAM-1, von Willebrand factor and Angiopoietin) were significantly elevated in sepsis patients as compared to healthy controls (P<0.01, **Table 3**), but there were no significant differences between the patient groups. Plasma levels of the proinflammatory cytokine IL-6 and the neutrophil chemokine, IL-8, were also significantly elevated in the sepsis patients (P<0.02, **Table 3**), but the elevations were not significantly different between the patient groups. Thus, while these plasma biomarkers can differentiate severally ill sepsis ICU patients from healthy controls, these biomarkers were not able to identify differences in neutrophil functional phenotypes in this sepsis patient group.

In contrast, NETs, an important component of the neutrophil response to infection, were significantly elevated in the plasma of Hyperimmune patients (P<0.003), but not in Hypoimmune or Hybrid patients (P=NS) as compared to controls (**Figure 7B**). Further, plasma NETs levels in Hyperimmune patients were significantly elevated as compared to either Hybrid or Hypoimmune patient plasma values (P<0.003). Thus, the Hyperimmune group, in contrast to the Hypoimmune and Hybrid groups, is characterized by elevated circulating neutrophils and enhanced plasma levels of NETs.

## Discussion

**Figure 8:**
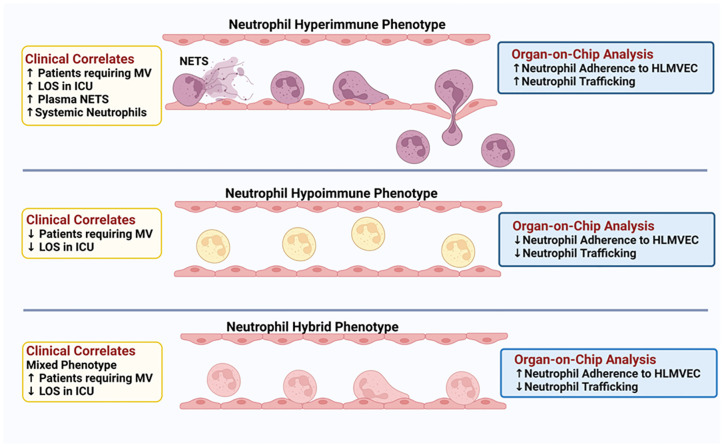
Schematic Illustration: Three distinct neutrophil functional phenotypes were identified in ICU sepsis patients by organ-on-chip analysis of neutrophil *ex vivo* adhesion and migration patterns. These functional neutrophil phenotypes had distinct clinical correlates.

A central component of sepsis is organ damage as a result of a dysregulated host response to infection (4) and the diverse immune response observed in sepsis patients may determine disease progression and outcome. To date, few studies have examined the functional responses of neutrophils in sepsis patients. Here, we demonstrate for the first time, functional phenotyping of neutrophils from sepsis patients revealing different attributes that impact neutrophil interactions with the vascular endothelium and trafficking into critical organs. Employing Organ-on-Chip analysis, we identified three neutrophil functional phenotypes in sepsis patients based on *ex vivo* adhesion and migration patterns across human lung endothelial cells. We further determined that these neutrophil functional phenotypes express unique *proteomic signatures* that correlated with important clinical parameters such as hypoxemia, mechanical ventilation, and length of stay in the ICU (**Figure 8**).

Using our Organ-on-Chip which integrates a microfluidic assay of the entire leukocyte adhesion cascade (31, 32, 34–36, 38, 61), we are able to evaluate the role of neutrophil functional phenotypes in neutrophil-endothelial cell interactions and neutrophil trafficking to determine how heterogeneous neutrophil subpopulations differentially impact vascular barrier disruption and neutrophil migration in a physiologically relevant *ex vivo* system. In this study, we found that different groups of sepsis patients in the ICU had distinct functional responses to activation in their adhesion to human primary pulmonary endothelial cells and their ability to migrate though endothelial cells in response to chemotactic stimuli. Based on *ex vivo* neutrophil functional studies in response to buffer or cytokine activation and encompassing multiple adherence (i.e. different shear rates and bifurcations) and kinetics of migration patterns into the tissue compartment, we identified three distinct phenotypes ranging from Hyperimmune to Hypoimmune in response to stimuli. The Hyperimmune patient group, which was the largest patient group, demonstrated the greatest *ex vivo* neutrophil activation based on neutrophil adhesion and migration, and had the greatest pulmonary dysfunction among the sepsis patients. This group had the most patients on mechanical ventilation and with higher FiO_2_ requirements. The cause of respiratory failure in this patient group was predominantly the result of hypoxia and ARDS. The Hyperimmune patients also had a significantly greater length of stay in the ICU as compared to the Hypoimmune and Hybrid patient groups. Furthermore, the Hyperimmune patient group, in contrast to the Hypoimmune and Hybrid groups, had significantly elevated circulating neutrophils. Further, the increased number of neutrophils was not related to increased release of immature neutrophils as there were no significant differences in blood levels of bands among the groups (**Table 3**). Finally, it should be noted that equal numbers of neutrophils were introduced into the Organ-on-Chip, so the observed increased adhesion and migration in the Hyperimmune group was not the result of higher neutrophil numbers. Thus, in the Hyperimmune group, not only were these neutrophils hyperactivated as evidenced by *ex vivo* increased adhesion and migration, but there may also be increased neutrophil release from the bone marrow or decreased neutrophil apoptosis in these patients, resulting in increased circulating neutrophils (62).

Interestingly, the Hypoimmune patient group with the suppressive *ex vivo* functional adhesion/migration phenotype did not have the most severe illness. Rather this group had fewer number of patients requiring mechanical ventilation and shorter length of stay in the ICU. The long-term clinical effects of this phenotype are not known and whether the decreased responsiveness to cytokine activation in this patient group makes them more susceptible to secondary infections is not known and requires further study. We also identified a Hybrid phenotype in a small group of patients that displayed increased adhesion but impaired migration suggesting when activated, these patietns' neutrophils may accumulate in the vascular compartment without trafficking into organs. This accumulation of activated neutrophils could lead to significant damage of the vascular endothelium. As shown in **Table 3**, there was a trend for higher plasma levels of angiopoietin, IL-6 and IL-8 in the Hybrid group, but due to the small sample size, it did not reach significance. Identification of these patient phenotypes could not have been possible based on clinical data alone as there were no significant differences in commonly measured clinical variables or demographics such as age, sex, ethnicity, source of infection, clinical laboratory values, Glasgow Coma Score or qSOFA assessment between the three patient groups identified in this study (**Table 2**, **Supplementary Table 4**). Thus, our study provides novel insights into distinct molecular phenotypes that are not simply explained by clinical parameters.

While omic analysis has been used previously to characterize the immune status of sepsis patients, there have been limited neutrophil functional studies and very few linking omic alterations to functional consequences (10, 12, 15–18, 63–67). Investigating the functionality of neutrophils during sepsis and correlating it to phenotypic omics (e.g., proteomic) analysis, is critical not only for a comprehensive understanding of the underlying molecular expression within the cells but also for determining how these changes can significantly affect immune function and clinical parameters. Using proteomic analysis, we determined that these three functional neutrophil phenotypes in sepsis patients have distinct “proteomic signatures” associated with expression of unique proteins that are phenotype specific. Importantly, we demonstrated significant differences in expression among the patient groups in neutrophil proteins involved in critical aspects of the septic response. Neutrophils with the Hyperimmune and Hybrid phenotype, compared to the Hypoimmune phenotype, had significantly increased expression of proteins associated with adherence, cytoskeleton and defense proteins involved in proinflammatory cell functions (**Figure 6**) and these distinct proteomic signatures are associated with significant neutrophil functional differences. Detection of unique protein expression in the different neutrophil phenotypes may help identify novel therapeutic targets for a specific neutrophil phenotype (67, 68).

Neutrophils are critical components of the innate immune system and play an important role in the elimination of invading pathogens through antimicrobial activities, as well as maintaining immune homeostasis (69). However, during sepsis and the development of immune dysregulation, multiple alterations in neutrophil function have been reported including delayed apoptosis resulting in uncontrolled activation and persistent neutrophil dysfunction, and increased NET formation (6–8, 20, 23, 24, 69, 70). During NETosis, neutrophils extrude strands of nuclear material (such as DNA) which form a web-like structure that is composed of decondensed chromatin fibers decorated with histones and antimicrobial proteins, such as neutrophil elastase, MPO and cathepsin (71). While NETs are critical components of the neutrophil bactericidal repertoire, dysregulated NETs release in sepsis can exacerbate inflammation and cause organ dysfunction (69, 72). NETs are particularly damaging to endothelial cells and have been shown to adhere to and activate the vascular endothelium during sepsis, contributing to local inflammation, increased neutrophil trafficking and damaging endothelial cells (69, 73). Elevated levels of NETs can shift endothelial cells to a pro-inflammatory and pro-coagulant phenotype leading to increased tissue damage and organ failure (70, 72). In our group of sepsis patients, only patients in the Hyperimmune group had significantly elevated plasma levels of NETs as compared to the Hypoimmune and Hybrid group. This elevation in plasma NETs was associated with increased systemic neutrophils in the Hyperimmune group. Further mechanistic studies are needed to ascertain whether the elevated NETs in the Hyperimmune group is the result of increased NETs production by neutrophils or simply the result of increased circulating neutrophils. Regardless of mechanism, this elevation in plasma NETs may be associated with disease severity as the Hyperimmune group was the most severely ill with significant pulmonary dysfunction, increased mechanical ventilation requirements, and increased length of stay in the ICU. The identification of elevated NETs in a specific phenotype may provide an important biomarker for the Hyperimmune group as well as suggesting a possible therapeutic approach for this group of patients.

In conclusion, we employed Organ-on-Chip technology to categorize septic patients into distinct neutrophil functional phenotypes that were associated with unique *proteomic signatures*. Importantly in this study, we also identified significant associations between these neutrophil phenotypes and clinical outcomes such as pulmonary function, mechanical ventilation and length of stay in the ICU. Sepsis is a heterogeneous disease and the host response in sepsis patients is highly diverse impacting immune function and response to infection. Identification of these distinct neutrophil phenotypes in sepsis patients may provide important insight into the failure of some sepsis drugs in clinical trials. Further, recognition of diverse functional neutrophil responses and protein expression may provide important insight for patient stratification in clinical trials and may identify novel therapeutic targets for specific sepsis patient populations. Finally, recognition of diverse functional neutrophil responses may help identify sepsis patients who would benefit from specific treatments, such as, for example, immunosuppressive therapies for patients with hyperactive immune response vs. those patients (e.g hypoactive group) who may be negatively impacted by immunosuppressive therapies as they are already immune suppressed. Thus, identification of distinct functional immune status in sepsis patients will provide important information for precision medicine and improving patient outcomes.

## Data availability statement

The datasets presented in this study can be found in online repositories. The names of the repository/repositories and accession number(s) can be found below: PXD041007 (PRIDE).

## Ethics statement

The studies involving humans were approved by Temple University Institutional Review Board (Temple University IRB protocol #24515). The studies were conducted in accordance with the local legislation and institutional requirements. The participants provided their written informed consent to participate in this study. Written informed consent was obtained from the individual(s) for the publication of any potentially identifiable images or data included in this article.

## Author contributions

QY: Data curation, Formal analysis, Writing – review and editing, Writing – original draft, Investigation, Visualization. JL: Data curation, Formal analysis, Writing – review and editing, Investigation, Writing – original draft, Visualization. RP: Investigation, Methodology, Writing – review and editing. SP: Methodology, Supervision, Writing – review and editing, Investigation. HZ: Formal analysis, Methodology, Visualization, Writing – review and editing. EP: Formal analysis, Investigation, Writing – review and editing. HE: Formal analysis, Investigation, Methodology, Visualization, Writing – review and editing. NDM: Formal analysis, Investigation, Methodology, Visualization, Writing – review and editing. CM: Investigation, Methodology, Writing – review and editing. SM: Data curation, Formal analysis, Methodology, Supervision, Writing – review and editing, Investigation, Visualization. NTM: Investigation, Methodology, Supervision, Writing – review and editing. BP: Conceptualization, Formal analysis, Investigation, Methodology, Supervision, Writing – review and editing. MK: Conceptualization, Data curation, Formal analysis, Funding acquisition, Investigation, Methodology, Project administration, Resources, Supervision, Writing – review and editing. LK: Conceptualization, Data curation, Formal analysis, Funding acquisition, Investigation, Methodology, Project administration, Resources, Supervision, Validation, Visualization, Writing – original draft, Writing – review and editing.

## Supplementary material

Supplementary Video 1 Fluorescently labeled Human neutrophils roll, adhere, and migrate on/across the activated primary human lung endothelial cells in our Organ-on-Chip.
