# Supplementary material for: Distinct functional neutrophil phenotypes in sepsis patients correlate with disease severity
Source: Front Immunol. 2024 Mar 8;15:1341752. doi: 10.3389/fimmu.2024.1341752 (PMC10957777; doi:10.3389/fimmu.2024.1341752)
Supplement: Supplementary file 1 [file Table_1.pdf]

Supplementary Tables

Supplementary Table 1

Commonly expressed upregulated proteins between the 3 sepsis phenotypes

Protein Symbol	Protein Class(es)/BP Functions	Protein Name
SAA1	Apolipoprotein	serum amyloid A1
UVRAG	Autophagy	UV radiation resistance associated
USF1	Cellular transcription factor	upstream transcription factor 1
TOR2A	Chaperone	torsin family 2 member A
COX17	Chaperone: cyctochrome c oxidase copper chaperone	cytochrome c oxidase copper chaperone COX17
BCL7C	Chromatin binding/regulatory/regulatory	BAF chromatin remodeling complex subunit BCL7C
EED	Chromatin binding/regulatory/regulatory	embryonic ectoderm development
NCAPG	Chromatin binding/regulatory/regulatory: Condensation of chromosomes	non-SMC condensin I complex subunit G
HEXIM1	Cyclin-dependent protein serine/threonine kinase inhibitor activity	HEXIM P-TEFb complex subunit 1
CNN3	Cytoskeletal	calponin 3
THAP11	DNA binding protein	THAP domain containing 11
LANCL2	DNA binding protein: small nuclear RNA processing	LanC like 2
VAMP5	Docking/fusion of vesicles	vesicle associated membrane protein 5
TSEN34	Endoribonuclease (trna-splicing endonuclease)	tRNA splicing endonuclease subunit 34
NACA	Gene specific transcriptional regulator: basic helix loop helix transcription factor	nascent polypeptide associated complex subunit alpha
ETV6	Gene specific transcriptional regulator: winged helix/forkhead transcription factor	ETS variant transcription factor 6
RGS10	GTPase activating	regulator of G protein signaling 10
ITGB3	Integrin beta; cell adhesion and endothelial cell migration	integrin subunit beta 3
MGAT2	Metabolite interconversion enzyme: acyltransferase	alpha-1,6-mannosyl-glycoprotein 2-beta-N-acetylglucosaminyltransferase
SDR39U1	Metabolite interconversion enzyme: epimerase/racemase	short chain dehydrogenase/reductase family 39U member 1
FAR1	Metabolite interconversion enzyme: fatty acid reduction	fatty acyl-CoA reductase 1
GNPDA2	Metabolite interconversion enzyme: isomerase	glucosamine-6-phosphate deaminase 2
IFI30	Metabolite interconversion enzyme: reductase	IFI30 lysosomal thiol reductase

TGM2	Metabolite interconversion enzyme: transferase	transglutaminase 2
RABL2B	Protein binding activity modulator: small GTPase	RAB, member of RAS oncogene family like 2B
UBL5	Protein degradation	ubiquitin like 5
ECI2	Protein modifying enzyme: hydratase/isomerase	enoyl-CoA delta isomerase 2
TFRC	Protein modifying enzyme: metalloprotease	transferrin receptor
CDK1	Protein modifying enzyme: non-receptor serine/threonine protease	cyclin dependent kinase 1
CPD	Protein modifying enzyme: protease	carboxypeptidase D
PTP4A2	Protein modifying enzyme: protein phosphatase	protein tyrosine phosphatase 4A2
SSH3	Protein modifying enzyme: protein phosphatase	slingshot protein phosphatase 3
TRAPPC12	Protein modifying enzyme: serine protease	trafficking protein particle complex subunit 12
AAK1	Protein modifying enzyme: Serine/threonine kinase	AP2 associated kinase 1
RAB6D	Protein modifying enzyme: small GTPase	RAB6D, member RAS oncogene family
INTS1	RNA binding	integrator complex subunit 1
XPOT	RNA metabolism protein: Exportin-t	exportin for tRNA
RTCA	RNA metabolism protein: RNA 3'-termina phosphatase cyclase)	RNA 3'-terminal phosphate cyclase
TRMT10A	RNA methyltransferase: t-rna methyltransferase	tRNA methyltransferase 10A
SRSF6	RNA splicing factor: serine/arginine rich splicing factor	serine and arginine rich splicing factor 6
SNX14	Scaffold	sorting nexin 14
CD74	Scaffold; neutrophil chemotaxis	CD74 molecule
FTH1	Storage	ferritin heavy chain 1
LARS2	Translational: aminoacyl-tRNA synthetase	leucyl-tRNA synthetase 2, mitochondrial
PABPN1	Translational: translation initiation factor	poly(A) binding protein nuclear 1
MTX2	Transporter	metaxin 2
SLC35A5	Transporter	solute carrier family 35 member A5
ABCB7	Transporter: ATP-binding cassette transporter	ATP binding cassette subfamily B member 7
ABCC12	Transporter: ATP-binding cassette transporter	ATP binding cassette subfamily C member 12
SLC39A11	Transporter: Secondary carrier transporter (zinc transporter)	solute carrier family 39 member 11

Commonly expressed downregulated proteins between the 3 sepsis phenotypes

Protein Symbol	Protein Class(es)/BP Functions	Protein Name
BCL10	Apoptosis	BCL10 immune signaling adaptor
CCT6B	Chaperone	chaperonin containing TCP1 subunit 6B
IFIT3	Defense/Immunity	interferon induced protein with tetratricopeptide repeats 3
SVIP	Endoplasmic reticulum degradation inhibitor	small VCP interacting protein
SNCA	Membrane traffic: alpha synuclein	synuclein alpha
TGOLN2	Membrane traffic: trans-Golgi network integral membrane protein 2	trans-golgi network protein 2
CMPK2	Metabolite interconversion enzyme (nucleotide kinase): UMP-CMP kinase 2	cytidine/uridine monophosphate kinase 2
NOX4	Metabolite interconversion enzyme (oxidase)	NADPH oxidase 4
PRG2	Metabolite interconversion enzyme (peroxidase): Peroxidasin homolog	proteoglycan 2, pro eosinophil major basic protein
PPP2CA	Protein modifying enzyme (protein phosphatase): serine/threonine protein phosphatase 1a	protein phosphatase 2 catalytic subunit alpha
CD200R1	Transmembrane signal receptor: Cell surface glycoprotein	CD200 receptor 1
LST1	Transporter: Solute carrier organic anion transporter	leukocyte specific transcript 1
