# Supplementary material for: Distinct functional neutrophil phenotypes in sepsis patients correlate with disease severity
Source: Front Immunol. 2024 Mar 8;15:1341752. doi: 10.3389/fimmu.2024.1341752 (PMC10957777; doi:10.3389/fimmu.2024.1341752)
Supplement: Supplementary file 2 [file Table_2.pdf]

Supplementary Table 2
Unique up and down regulated proteins within the different phenotypes

<u>Hyperimmune Unique Upregulated Proteins</u>

Protein Symbol	Protein Class(es)/BP Functions	Protein Name
HSP90AA2P	Chaperone	heat shock protein 90 alpha family class A member 2, pseudogene
H2AC21	Chromatin binding/regulatory	H2A clustered histone 21
NCAPH	Chromatin binding/regulatory	non-SMC condensin I complex subunit H
POLD1	DNA metabolism	DNA polymerase delta 1, catalytic subunit
HSBP1	Gene specific transcription regulator: heat shock response	heat shock factor binding protein 1
DPY19L3	Metabolite interconversion enzyme: transferase	dpy-19 like C-mannosyltransferase 3
TSTD1	Metabolite interconversion enzyme: transferase	thiosulfate sulfurtransferase like domain containing 1
VWA5A	Nucleoplasm-function related	von Willebrand factor A domain containing 5A
TBC1D24	Protein binding activity modulator: GTPase activating protein	TBC1 domain family member 24
GNAI2	Protein binding activity modulator: heterotrimeric G protein	G protein subunit alpha i2
PPT1	Protein modifying enzyme	palmitoyl-protein thioesterase 1
SNX15	Protein modifying enzyme: non receptor serine/threonine protein kinase	sorting nexin 15
PRSS1	Protein modifying enzyme: serine protease	serine protease 1
PRTN3	Protein modifying enzyme: serine protease; neutrophil extravasation	proteinase 3
CAB39L	Protein modifying enzyme: serine/threonine kinase activity	calcium binding protein 39 like
DDX10	RNA metabolism	DEAD-box helicase 10
LRSAM1	Scaffold: E3 ubiquitin-protein ligase	leucine rich repeat and sterile alpha motif containing 1
MRPL12	Translational: ribosomal protein	mitochondrial ribosomal protein L12

JAGN1	Transmembrane signal receptor; neutrophil-mediated immunity	jagunal homolog 1

Hyperimmune Unique Downregulated Proteins

Protein Symbol	Protein Class(es)/BP Functions	Protein Symbol
ANTXR2	Cell adhesion: ECM	Anthrax toxin receptor 2
WDR11	Cell cycle	WD repeat domain 11
H2BC13	Chromatin binding/regulatory	H2B clustered histone 13
SP110	Chromatin binding/regulatory	SP110 nuclear body protein
RWDD1	Cytoplasmic translation	RWD domain containing 1
RWDD1	cytoplasmic translation	RWD domain containing 1
INF2	Cytoskeletal: actin	inverted formin 2
TGFB1I1	Cytoskeletal: actin binding cytoskeletal	transforming growth factor beta 1 induced transcript 1
ADD2	Cytoskeletal: non motor actin binding	adducin 2
IGHG4	Defense/immunity	immunoglobulin heavy constant gamma 4 (G4m marker)
RETN	Defense/immunity	resistin
RSAD2	Defense/immunity	radical S-adenosyl methionine domain containing 2
NKAP	Gene specific transcription regulator: activation of nfkb	NFKB activating protein
RYR2	Ion (calcium) channel	ryanodine receptor 2
MBOAT7	Metabolite interconversion enzyme: acetyltransferase	membrane bound O-acyltransferase domain containing 7
OLAH	Metabolite interconversion enzyme: esterase	oleoyl-ACP hydrolase
DNPH1	Metabolite interconversion enzyme: hydrolase	2'-deoxynucleoside 5'-phosphate N-hydrolase 1
TTR	Metabolite interconversion enzyme: hydrolase	transthyretin
TMEM179B	Nucleolus function related	transmembrane protein 179B
CYB561D2	Protein binding activity modulator: heme binding	cytochrome b561 family member D2
GNAO1	Protein binding activity modulator: heterotrimeric G protein	G protein subunit alpha o1
CDC42	Protein binding activity modulator: small GTPase	cell division cycle 42
SPPL2A	Protein modifying enzyme: aspartic protease	signal peptide peptidase like 2A
GGT2	Protein modifying enzyme: protease	gamma-glutamyltransferase 2
CELF1	RNA metabolism	CUGBP Elav-like family member 1
CRNKL1	RNA splicing factor	crooked neck pre-mRNA splicing factor 1
SIGLEC14	Sialic acid binding	sialic acid binding Ig like lectin 14
HBG2	Transfer: globin	hemoglobin subunit gamma 2

EPHA2	Transmembrane signal receptor	EPH receptor A2
AQP1	Transport	aquaporin 1 (Colton blood group)
TOMM40	Transport	translocase of outer mitochondrial membrane 40

Hypoimmune Unique Upregulated Proteins

Protein Symbol	Protein Class(es) /BP Functions	Protein Name
EGFLAM	Calcium ion binding	EGF like, fibronectin type III and laminin G domains
ITGA2B	Integrin alpha; cell adhesion; cell-matrix adhesion	integrin subunit alpha 2b
HSPB1	Chaperone	heat shock protein family B (small) member 1
SURF1	Chaperone	SURF1 cytochrome c oxidase assembly factor
CARM1	Chromatin binding/regulatory	coactivator associated arginine methyltransferase 1
CIAO2B	Chromatin binding/regulatory	cytosolic iron-sulfur assembly component 2B
ELP4	Chromatin binding/regulatory	elongator acetyltransferase complex subunit 4
CTTN	Cytoskeletal; adherens-type junctions	cortactin
PLS3	Cytoskeletal: non motor binding	plastin 3
MAP1B	Cytoskeletal: non motor microtubule	microtubule associated protein 1B
CARD8	Defense/immunity	caspase recruitment domain family member 8
MCM6	DNA metabolism	minichromosome maintenance complex component 6
SPARC	ECM; endothelial cell migration; endothelial cell proliferation	secreted protein acidic and cysteine rich
LGALS8	ECM	galectin 8
HBQ1	Human fetal erythroid tissue function	hemoglobin subunit theta 1
LRP10	Ldl receptor family	LDL receptor related protein 10
UAP1	Metabolism interconversion enzyme: Udp-n-acetylglucosamine biosynthetic process	UDP-N-acetylglucosamine pyrophosphorylase 1
AMDHD2	Metabolite interconversion enzyme: deacetylase	amidohydrolase domain containing 2
NDUFV2	Metabolite interconversion enzyme: dehydrogenase	NADH:ubiquinone oxidoreductase core subunit V2
DNPH1	Metabolite interconversion enzyme: hydrolase	2'-deoxynucleoside 5'-phosphate N-hydrolase 1
IDI1	Metabolite interconversion enzyme: isomerase	isopentenyl-diphosphate delta isomerase 1

METTL7A	Metabolite interconversion enzyme: methyltransferase	methyltransferase like 7A
UQCRQ	Metabolite interconversion enzyme: oxidoreductase	ubiquinol-cytochrome c reductase complex III subunit VII
CYB5R3	Metabolite interconversion enzyme: reductase	cytochrome b5 reductase 3
PYCR3	Metabolite interconversion enzyme: reductase	pyrroline-5-carboxylate reductase 3
CSNK2B	Protein binding activity modulator	casein kinase 2 beta
MLKL	Protein binding activity modulator	mixed lineage kinase domain like pseudokinase
NRGN	Protein binding activity modulator: calmodulin binding	neurogranin
RALA	Protein binding activity modulator: small GTPase	RAS like proto-oncogene A
PLOD1	Protein modifying enzyme: hydroxylation of lysyl residues in collagen peptides	procollagen-lysine,2-oxoglutarate 5-dioxygenase 1
CTSL	Protein modifying enzyme: lysosomal cysteine proteinase in catabolism	cathepsin L
CDK11B	Protein modifying enzyme: serine/threonine kinase	cyclin dependent kinase 11B
PPP1R3D	Protein modifying enzyme: serine/threonine phosphorylation	protein phosphatase 1 regulatory subunit 3D
THUMPD1	RNA binding	THUMP domain containing 1
MBNL1	RNA metabolism	muscleblind like splicing regulator 1
RBM3	RNA metabolism	RNA binding motif protein 3
RBMXL1	RNA metabolism	RBMX like 1
SF3B6	RNA metabolism	splicing factor 3b subunit 6
SNRPA	RNA metabolism	small nuclear ribonucleoprotein polypeptide A
MMRN1	Scaffold	multimerin 1
VIPAS39	Sorting: lysosomal proteins	VPS33B interacting protein, apical-basolateral polarity regulator, spe-39 homolog
EIF3K	Translational: translation initiation factor	eukaryotic translation initiation factor 3 subunit K

EIF4G3	Translational: translation initiation factor	eukaryotic translation initiation factor 4 gamma 3
SLC3A2	Transmembrane signal receptor	solute carrier family 3 member 2
EMB	Transmembrane signal receptor: glycoprotein	embigin
GYPC	Transmembrane signal receptor: glycoprotein	glycophorin C (Gerbich blood group)
CD9	Transmembrane signal receptor: glycoprotein	CD9 molecule
RAB27B	Transmembrane signal receptor: vesicular fusion and trafficking	RAB27B, member RAS oncogene family
TM9SF2	Transmembrane 9 superfamily	transmembrane 9 superfamily member 2
TRPV2	Transmembrane signal receptor: ion channel	transient receptor potential cation channel subfamily V member 2
TANGO2	Transport	transport and golgi organization 2 homolog

<u>Hypoimmune Unique Downregulated Proteins</u>

Protein Symbol	Protein Class(es)/BP Functions	Protein Name
GDAP2	Retinoic acid function	ganglioside induced differentiation associated protein 2
TNFAIP8	Apoptosis	TNF alpha induced protein 8
WDFY4	Autophagy	WDFY family member 4
CLEC12A	C-type lectin family	C-type lectin domain family 12 member A
CLEC16A	C-type lectin family	C-type lectin domain containing 16A
JAML	Cell adhesion; neutrophil extravasation	junction adhesion molecule like
SIGLEC9	Sialic acid binding; cell adhesion	sialic acid binding Ig like lectin 9
DNAJC1	Chaperone	DnaJ heat shock protein family (Hsp40) member C1
GET4	Chaperone	guided entry of tail-anchored proteins factor 4
TTC37	Chaperone	tetratricopeptide repeat domain 37
ISG15	Chemotaxis	ISG15 ubiquitin like modifier
CBX1	Chromatin binding/regulatory	chromobox 1
NIPBL	Chromatin binding/regulatory	NIPBL cohesin loading factor
PAF1	Chromatin binding/regulatory	PAF1 homolog, Paf1/RNA polymerase II complex component
SMARCD2	Chromatin binding/regulatory	SWI/SNF related, matrix associated, actin dependent regulator of chromatin, subfamily d, member 2
SIRT5	Chromatin binding/regulatory	sirtuin 5
ERGIC1	Cycling membrane	endoplasmic reticulum-golgi intermediate compartment 1
EML3	Cytoskeletal binding: microtubule binding	EMAP like 3
JPT2	Cytoskeletal: microtubule binding	Jupiter microtubule associated homolog 2
PCLO	Cytoskeletal: presynaptic cytoskeletal matrix	piccolo presynaptic cytomatrix protein
IFIT2	Defense/immunity	interferon induced protein with tetratricopeptide repeats 2
HDDC2	DNA binding	HD domain containing 2
POLD1	DNA metabolism	DNA polymerase delta 1, catalytic subunit
TERF2IP	DNA metabolism	TERF2 interacting protein
HMGA1	DNA metabolism: endodeoxyribonuclease	high mobility group AT-hook 1
CLC	ECM	Charcot-Leyden crystal galectin
PRG3	ECM	proteoglycan 3, pro eosinophil major basic protein 2
TNFAIP6	ECM: hyaluronan-binding; cell adhesion	TNF alpha induced protein 6
WDR61	Gene specific transcriptional regulation	WD repeat domain 61
WTAP	Gene specific transcriptional regulation	WT1 associated protein
HES1	Gene specific transcriptional	hes family bHLH transcription factor 1

	regulator: basic helix loop helix transcription factor	
XPO6	Importin beta	exportin 6
ALDH18A1	Metabolite interconversion enzyme: dehydrogenase	aldehyde dehydrogenase 18 family member A1
HIBADH	Metabolite interconversion enzyme: dehydrogenase	3-hydroxyisobutyrate dehydrogenase
ALG1	Metabolite interconversion enzyme: glycosyltransferase	ALG1 chitobiosyldiphosphodolichol beta-mannosyltransferase
FAHD1	Metabolite interconversion enzyme: hydrolase	fumarylacetoacetate hydrolase domain containing 1
NDUFA2	Metabolite interconversion enzyme: oxidoreductase	NADH:ubiquinone oxidoreductase subunit A2
ALOX15	Metabolite interconversion enzyme: oxygenase	arachidonate 15-lipoxygenase
BLVRB	Metabolite interconversion enzyme: reductase	biliverdin reductase B
DHCR7	Metabolite interconversion enzyme: reductase	7-dehydrocholesterol reductase
AASDHPPT	Metabolite interconversion enzyme: transferase	aminoadipate-semialdehyde dehydrogenase-phosphopantetheinyl transferase
CERS2	Metabolite interconversion enzyme: transferase	ceramide synthase 2
GFM1	Mitochondrial translation elongation factor	G elongation factor mitochondrial 1
PMPCA	Protein modifying enzyme: metalloprotease	peptidase, mitochondrial processing subunit alpha
BAZ1B	Protein modifying enzyme: non receptor tyrosine protein kinase	bromodomain adjacent to zinc finger domain 1B
PCNP	Protein modifying enzyme: proteasome-mediated, ubiquitin-dependent catabolic process and ubiquitination	PEST proteolytic signal containing nuclear protein

PDP1	Protein modifying enzyme: protein phosphatase	pyruvate dehydrogenase phosphatase catalytic subunit 1
SSH2	Protein modifying enzyme: protein phosphatase	slingshot protein phosphatase 2
SSH2	Protein modifying enzyme: protein phosphatase	slingshot protein phosphatase 2
GZMH	Protein modifying enzyme: serine protease	granzyme H
GZMH	Protein modifying enzyme: serine protease	granzyme H
NELFE	RNA binding	negative elongation factor complex member E
PRPF38B	RNA binding	pre-mRNA processing factor 38B
SLIRP	RNA binding	SRA stem-loop interacting RNA binding protein
NELFE	RNA binding	negative elongation factor complex member E
PRPF38B	RNA binding	pre-mRNA processing factor 38B
SLIRP	RNA binding	SRA stem-loop interacting RNA binding protein
DHX36	RNA metabolism	DEAH-box helicase 36
RNASE3	RNA metabolism	ribonuclease A family member 3
SH2D3C	Scaffold	SH2 domain containing 3C
MRPL49	Translational: ribosomal	mitochondrial ribosomal protein L49
MRPS17	Translational: ribosomal	mitochondrial ribosomal protein S17
EIF2B3	Translational: translation initiation factor	eukaryotic translation initiation factor 2B subunit gamma
DENR	Translational: translational initiation factor	density regulated re-initiation and release factor
EXOC6	Transmembrane signal receptor	exocyst complex component 6
MX1	Transmembrane signal receptor	MX dynamin like GTPase 1
RUSF1	Transmembrane signal receptor	RUS family member 1
VPS36	Transmembrane signal receptor	vacuolar protein sorting 36 homolog
MIA3	Transmembrane signal receptor; leukocyte cell-cell adhesion and migration	MIA SH3 domain ER export factor 3
GHITM	Transport	growth hormone inducible transmembrane protein
PITPNM1	Transport	phosphatidylinositol transfer protein membrane associated 1
TBC1D23	Transport	TBC1 domain family member 23
SEC13	Transport	SEC13 homolog, nuclear pore and COPII coat complex component

Hybrid Unique Upregulated Proteins

Protein Symbol	Protein Class(es) /BP Function	Protein Name
PIN1	Chaperone	peptidylprolyl cis/trans isomerase, NIMA-interacting 1
MYH11	Cytoskeletal: actin binding	myosin heavy chain 11
IGKV3D-11	Defense/immunity: antibody	immunoglobulin kappa variable 3D-11
IGHG1	Defense/immunity: antibody receptor	immunoglobulin heavy constant gamma 1 (G1m marker)
VTN	ECM	vitronectin
NR2C2	Gene specific transcription regulator: C4 zinc finger nuclear receptor	nuclear receptor subfamily 2 group C member 2
WNT7A	Intercellular signaling	Wnt family member 7A
IDH3A	Metabolite interconversion enzyme: dehydrogenase	isocitrate dehydrogenase (NAD(+)) 3 catalytic subunit alpha
SUMO3	Protein binding activity: sumoylation	small ubiquitin like modifier 3
GGH	Protein modifying enzyme: cysteine protease	gamma-glutamyl hydrolase
ASRGL1	Protein modifying enzyme: protease	asparaginase and isoaspartyl peptidase 1
DDX24	RNA metabolism	DEAD-box helicase 24
RPL31	Translational: ribosomal	ribosomal protein L31
RPS20	Translational: ribosomal	ribosomal protein S20
ADGRE2	Transmembrane signal receptor	adhesion G protein-coupled receptor E2
FOLR3	Transmembrane signal receptor	folate receptor gamma

Hybrid Unique Downregulated Proteins

Protein Symbol	Protein Class(es)/BP Function	Protein Name
NINJ1	Cell adhesion; leukocyte chemotaxis	ninjurin 1
ANP32B	Chromatin binding/regulatory	acidic nuclear phosphoprotein 32 family member B
FLNC	Cytoskeletal: actin binding	filamin C
C9	Defense/immunity	complement C9
CD101	Defense/immunity	CD101 molecule

MAVS	Defense/immunity	mitochondrial antiviral signaling protein
HPS6	Organelle biogenesis	HPS6 biogenesis of lysosomal organelles complex 2 subunit 3
TBC1D24	Protein binding activity modulator: GTPase activating protein	TBC1 domain family member 24
PPP1R12C	Protein binding activity modulator: phosphatase modulator	protein phosphatase 1 regulatory subunit 12C
APP	Protein binding activity modulator: protease inhibitor	amyloid beta precursor protein
ECE1	Protein modifying enzyme: metalloprotease	endothelin converting enzyme 1
PAK1	Protein modifying enzyme: non-receptor serine/threonine protein kinase	p21 (RAC1) activated kinase 1
TAOK1	Protein modifying enzyme: non-receptor serine/threonine protein kinase	TAO kinase 1
GIGYF2	Protein modifying enzyme: tyrosine kinase receptor signaling	GRB10 interacting GYF protein 2
DCAF11	Protein modifying enzyme: ubiquitin protein ligase	DDB1 and CUL4 associated factor 11
UBE4A	Protein modifying enzyme: ubiquitin-protein ligase	Ubiquitin factor E4A
VIRMA	RNA binding	vir like m6A methyltransferase associated
ALB	Transfer	albumin
APOA2	Transfer: apolipoprotein	apolipoprotein A2
APOB	Transfer: apolipoprotein	apolipoprotein B
APOA1	Transfer: apolipoprotein	apolipoprotein A1
IL6R	Transmembrane signal receptor; Leukocyte chemotaxis	interleukin 6 receptor
TF	Transfer	transferrin
TMEM120A	Transmembrane signal receptor: ion channel	transmembrane protein 120A
ATP2B1	Transport: primary active transporter	ATPase plasma membrane Ca2+ transporting 1
LETM1	Transport: secondary carrier transporter	leucine zipper and EF-hand containing transmembrane protein 1
SEC62	Transport: ER translocation	SEC62 homolog, preprotein translocation factor
