# Supplementary material for: Distinct functional neutrophil phenotypes in sepsis patients correlate with disease severity
Source: Front Immunol. 2024 Mar 8;15:1341752. doi: 10.3389/fimmu.2024.1341752 (PMC10957777; doi:10.3389/fimmu.2024.1341752)
Supplement: Supplementary file 3 [file Table_3.pdf]

Supplementary Table 3
Common Proteins Shared Between 2 Phenotypes

Common Hyperimmune and Hypoimmune upregulated Proteins

Protein Symbol	Protein Class(es) /BP Functions	Protein Name
NINJ1	Homophilic adhesion; Leukocyte chemotaxis	ninjurin 1
TSPO	Steroid hormone synthesis	translocator protein

Common Hyperimmune and Hypoimmune Downregulated Proteins

Protein Symbol	Protein Class(es) /BP Functions	Protein Name
EPX	Metabolite interconversion enzyme	eosinophil peroxidase
THOC5	RNA binding	THO complex 5

Common Hypoimmune and Hybrid Upregulated Proteins

Protein Symbol	Protein Class(es) /BP Functions	Protein Name
FGG	Blood coagulation; cell-matrix adhesion	fibrinogen gamma chain
COMMD1	Copper homeostasis	copper metabolism domain containing 1
FNBP1L	Cytoskeletal: non-motor actin binding	formin binding protein 1 like
IGKC	Defense/immunity	immunoglobulin kappa constant
LBP	Defense/immunity; Leukocyte chemotaxis	lipopolysaccharide binding protein
FADS2	Metabolite interconversion enzyme: oxidoreductase	fatty acid desaturase 2
TIGAR	Metabolite interconversion enzyme: phosphatase	TP53 induced glycolysis regulatory phosphatase
SERPINH1	Protein binding activity modulator: protease inhibitor	serpin family H member 1
MRPL1	Translational: ribosomal	mitochondrial ribosomal protein L1

FPR1	Transmembrane signal receptor: G-protein coupled receptor	formyl peptide receptor 1

Common Hypoimmune and Hybrid Downregulated Proteins

Protein Symbol	Protein Class(es)/BP Functions	Protein Name
ORM2	Acute phase reactant	orosomucoid 2
OXR1	Metabolite interconversion enzyme: oxidoreductase activity	oxidation resistance 1
SULT1A1	Metabolite interconversion enzyme: transferase	sulfotransferase family 1A member 1
BIN1	Nucleocytoplasmic adapter	bridging integrator 1
MME	Protein modifying enzyme: metalloprotease	membrane metalloendopeptidase
DDX3Y	RNA metabolism: RNA helicase	DEAD-box helicase 3 Y-linked
RANBP9	Scaffold	RAN binding protein 9
ANO5	Transporter	anoctamin 5

Common Hyperimmune and Hybrid Upregulated Proteins

Protein Symbol	Protein Class(es)/BP Functions	Protein Name
EI24	Apoptosis	EI24 autophagy associated transmembrane protein
SUMO2	Apoptosis	small ubiquitin like modifier 2
CEACAM1	Cell adhesion	CEA cell adhesion molecule 1
CENPN	Cell cycle	centromere protein N
PPIH	Chaperone	peptidylprolyl isomerase H
SDF2L1	Chaperone	stromal cell derived factor 2 like 1
SGTB	Chaperone	small glutamine rich tetratricopeptide repeat co-chaperone beta
SMARCD2	Chromatin binding/regulatory/regulatory: chromatin regulatory	SWI/SNF related, matrix associated, actin dependent regulator of chromatin, subfamily d, member 2
KDM4B	Chromatin binding/regulatory/regulatory: histone modifying enzyme	lysine demethylase 4B
AFTPH	Clathrin binding activity	aftiphilin
CST7	Cystine protease inhibitor	cystatin F
DBN1	Cytoplasmic actin-binding protein	drebrin 1
TRIOBP	Cytoskeletal: actin binding cytoskeletal	TRIO and F-actin binding protein

ABRACL	Cytoskeletal: actin-filament process	ABRA C-terminal like
DYNC1LI2	Cytoskeletal: microtubules binding cytoskeletal	dynein cytoplasmic 1 light intermediate chain 2
MYO5C	Cytoskeletal: microtubules binding, actin binding motor	myosin VC
TPT1	Cytoskeletal: non motor microtubule binding	tumor protein, translationally-controlled 1
TUBA1C	Cytoskeletal: tubulin	tubulin alpha 1c
IFITM1	Defense/immunity	interferon induced transmembrane protein 1
PLTP	Defense/immunity	phospholipid transfer protein
IFRD1	Defense/Immunity: antiviral protein	interferon related developmental regulator 1
CCDC25	DNA binding activity; cell motility	coiled-coil domain containing 25
ZFHX4	DNA binding; RNA polymerase binding	zinc finger homeobox 4
HAPLN1	ECM	hyaluronan and proteoglycan link protein 1
SH3BGRL3	GTPase activator activity	SH3 domain binding glutamate rich protein like 3
MYADM	Heterotypic cell-cell adhesion	myeloid associated differentiation marker
C16orf54	Membrane component	chromosome 16 open reading frame 54
MIF	Metabolite interconversion enzyme: decarboxylase	macrophage migration inhibitory factor
PDHA1	Metabolite interconversion enzyme: dehydrogenase	pyruvate dehydrogenase E1 subunit alpha 1
ALG1	Metabolite interconversion enzyme: glycosyltransferase	ALG1 chitobiosyldiphosphodolichol beta-mannosyltransferase
PIP4K2C	Metabolite interconversion enzyme: kinase	phosphatidylinositol-5-phosphate 4-kinase type 2 gamma
DAGLB	Metabolite interconversion enzyme: lipase	diacylglycerol lipase beta
NPL	Metabolite interconversion enzyme: lyase	N-acetylneuraminate pyruvate lyase
NDUFB3	Metabolite interconversion enzyme: oxidoreductase	NADH:ubiquinone oxidoreductase subunit B3
ALOX12B	Metabolite interconversion enzyme: oxygenase	arachidonate 12-lipoxygenase, 12R type
ALPL	Metabolite interconversion enzyme: phosphatase	alkaline phosphatase, biomineralization associated
OXCT2	Metabolite interconversion enzyme: transferase	3-oxoacid CoA-transferase 2
SULT1A4	Metabolite interconversion enzyme: transferase	sulfotransferase family 1A member 4
TBC1D8	Protein binding activity modulator: GTPase-activating protein	TBC1 domain family member 8
GNG5	Protein binding activity modulator: heterotrimeric G-protein	G protein subunit gamma 5
RAB33A	Protein binding activity modulator: small GTPase	RAB33A, member RAS oncogene family

USP25	Protein modifying activity modulator: cysteine protease	ubiquitin specific peptidase 25
TPP1	Protein modifying activity modulator: serine protease	tripeptidyl peptidase 1
SRSF10	RNA metabolism: RNA splicing factor	serine and arginine rich splicing factor 10
INTS5	RNA polymerase binding	integrator complex subunit 5
TXLNA	Syntaxin binding	taxilin alpha
RPL34	Translational: ribosomal	ribosomal protein L34
RPL9	Translational: ribosomal	ribosomal protein L9
RPS29	Translational: ribosomal	ribosomal protein S29
TLR1	Transmembrane signal receptor	toll like receptor 1
SEH1L	Transporter	SEH1 like nucleoporin
CUTA	Transporter: primary active transporter	cutA divalent cation tolerance homolog
C7orf25	Undetermined	chromosome 7 open reading frame 25

Common Hyperimmune and Hybrid Downregulated Proteins

Protein Symbol	Protein Class(es) /BP Functions	Protein Name
ATG16L2	Apoptosis	autophagy related 16 like 2
UQCC2	Chaperone	ubiquinol-cytochrome c reductase complex assembly factor 2
H1-4	Chromatin binding/regulatory	H1.4 linker histone, cluster member
RBL2	Chromatin binding/regulatory	RB transcriptional corepressor like 2
CAP2	Cytoskeletal: actin binding	cyclase associated actin cytoskeleton regulatory protein 2
NDE1	Cytoskeletal: microtubule organization	nudE neurodevelopment protein 1
CRISP3	Defense/immunity	cysteine rich secretory protein 3
NLRP1	Defense/immunity	NLR family pyrin domain containing 1
DEFA1	Defense/immunity: antimicrobial response	defensin alpha 1
CAMP	Defense/immunity: antimicrobial response; neutrophil activation	cathelicidin antimicrobial peptide
IGSF6	Defense/immunity: immunoglobulin	immunoglobulin superfamily member 6
ATAD3B	DNA binding	ATPase family AAA domain containing 3B
SRC	Embryonic development	SRC proto-oncogene, non-receptor tyrosine kinase
MVB12B	Endosomal protein sorting	multivesicular body subunit 12B
HSD3B2	Metabolite interconversion	hydroxy-delta-5-steroid dehydrogenase, 3 beta- and steroid delta-isomerase 2

	enzyme: dehydratase	
CRYL1	Metabolite interconversion enzyme: dehydrogenase	crystallin lambda 1
UGCG	Metabolite interconversion enzyme: glycosyltransferase	UDP-glucose ceramide glucosyltransferase
METTL7A	Metabolite interconversion enzyme: methyltransferase	methyltransferase like 7A
PTGS1	Metabolite interconversion enzyme: oxygenase	prostaglandin-endoperoxide synthase 1
GSTM2	Metabolite interconversion enzyme: transferase	glutathione S-transferase mu 2
SYMPK	Polyadenylation regulation;	symplekin scaffold protein
GNB1	Protein binding activity modulator: heterotrimeric G-protein	G protein subunit beta 1
RAB6C	Protein binding activity modulator: small GTPase	RAB6C, member RAS oncogene family
UBE2L3	Protein modifying enzyme: ubiquitin-protein ligase	ubiquitin conjugating enzyme E2 L3
C9orf64	RNA binding: tRNA-guanine transglycosylation	chromosome 9 open reading frame 64
LACTB2	RNA metabolism: endoribonuclease	lactamase beta 2
COPS8	Scaffold	COP9 signalosome subunit 8
PLP2	Scaffold	proteolipid protein 2
RASSF3	Scaffold	Ras association domain family member 3
RPS5	Translational: ribosomal	ribosomal protein S5
PDCD4	Translational: translation factor	programmed cell death 4
SSR4	Transmembrane signal receptor: G-protein coupled receptor	signal sequence receptor subunit 4
EXOC8	Transport: vesicular trafficking	exocyst complex component 8

TMBIM1	Transporter: ion channel	transmembrane BAX inhibitor motif containing 1
CACNA1G	Transporter: voltage-gated ion channel	calcium voltage-gated channel subunit alpha1 G
