# Supplementary material for: Distinct functional neutrophil phenotypes in sepsis patients correlate with disease severity
Source: Front Immunol. 2024 Mar 8;15:1341752. doi: 10.3389/fimmu.2024.1341752 (PMC10957777; doi:10.3389/fimmu.2024.1341752)
Supplement: Supplementary file 4 [file Table_4.pdf]

Supplementary Table 4- Patient Characteristics of the Different Neutrophil Phenotypes

Sepsis Phenotype	Hypoimmune	Hyperimmune	Hybrid	P value
Number of Subjects	N=14	N=23	N=8	
Age (Years) (Mean±SD)<br>Range	56±6<br>24-88	60±3<br>29-81	49±7<br>26-71	NS
Gender N (% Male)	8 (57%)	10 (44%)	6 (75%)	NS
Ethnic Group N (%)				NS
White	6 (43%)	7 (30%)	5 (63%)	
Black	6 (43%)	6 (26%)	2 (25%)	
Hispanic	2 (14%)	8 (35%)	0 (0%)	
Asian	0 (0%)	2 (9%)	1 (13%)	
Type of Infection N (%)				NS
Gram negative bacteria	4 (29%)	9 (39%)	3 (38%)	
Gram Positive bacteria	4 (29%)	3 (13%)	3 (38%)	
Mixed organisms	2 (14%)	6 (26%)	2 (25%)	
Fungal	1 (7%)	0 (0%)	0 (0%)	
Organism negative	3 (21%)	5 (22%)	0 (0%)	
Source of Infection N(%)				NS
Pulmonary	8 (57%)	12 (52%)	4 (50%)	
UTI	3 (21%)	5 (22%)	0 (0%)	
Abdomen	2 (14%)	2 (9%)	2 (25%)	
Blood	1 (7%)	2 (9%)	2 (25%)	
Soft Tissue	0 (0%)	2 (9%)	0 (0%)	
Clinical Values (Mean±SD)				NS
Heart Rate	100±20	92±23	99±27	
Respiratory Rate	25±6	24±7	19±4	
CO_2	22±5	23±5	26±8	
O_2 Sat	97±3	97±3	97±5	
PaO_2	38±13	43±14	50±26	
Laboratory Values (Mean±SD)				NS
Glucose	183±138	193±151	163±70	
pH	7.4±0.1	7.3±0.1	7.3±0.2	
Albumin	3±1	2±1	3±1	
Total Protein	7±1	7±1	7±2	
Na	138±6	138±8	136±5	
K	4±1	4±1	4±1	

**Cl**	**107±9**	**104±10**	**102±8**
**WBC ×10^9/L**	**13±6**	**16±4**	**16±8**
**Hgb**	**11±3**	**10±2**	**10±2**
**Hct**	**31±8**	**31±7**	**30±7**
**Plt**	**181±84**	**197±111**	**171±132**
**Bands**	**1±2**	**3±12**	**1±2**

Characteristics of the Different Sepsis Neutrophil Phenotypes Abbreviations: UTI= urinary tract infection, CO_2=carbon dioxide, O_2 Sat=blood oxygen saturation, PaO_2= partial pressure of oxygen in the arterial blood, Na=sodium, K=potassium, Cl=chloride, WBC= white blood cells, Hgb=hemoglobin, Hct=hematocrit, Plt=platelet count, Bands=young (immature) neutrophils
